# The Biomechanical, Biochemical, and Morphological Properties of 31 Human Cadaveric Upper Limb Tendons: An Open Access Data Set

**DOI:** 10.1177/23259671261425673

**Published:** 2026-04-02

**Authors:** Carina L. Blaker, Dylan M. Ashton, David Chang, David O’Briain, Ying Liu, Samantha A. Hefferan, Nicholas Hartnell, Christopher B. Little, Elizabeth C. Clarke

**Affiliations:** * Murray Maxwell Biomechanics Laboratory, Institute of Bone and Joint Research, Kolling Institute, Northern Sydney Local Health District, Faculty of Medicine and Health, University of Sydney, St. Leonards, New South Wales, Australia; † Sydney School of Veterinary Science, Faculty of Science, The University of Sydney, Sydney, New South Wales, Australia; ‡ The University of Sydney, Sydney Musculoskeletal Health, Sydney, New South Wales, Australia; § Department of Hand Surgery, Royal North Shore Hospital, Northern Sydney Local Health District, Sydney, New South Wales, Australia; ‖ University Hospital Waterford, Waterford City, Waterford, Ireland; ¶ UPMC Whitfield, Waterford, Ireland; # University College Dublin School of Medicine, Dublin, Ireland; ** Raymond Purves Bone and Joint Research Laboratories, Institute of Bone and Joint Research, Kolling Institute, Northern Sydney Local Health District, Faculty of Medicine and Health, University of Sydney, St. Leonards, New South Wales, Australia; †† Bone Ligament and Tendon Pty Ltd, Bowral, New South Wales, Australia; Investigation performed at the Kolling Institute, Northern Sydney Local Health District, University of Sydney, St. Leonards, New South Wales, Australia

**Keywords:** autograft, allograft, hand, wrist, elbow, shoulder, flexor, extensor, rotator cuff

## Abstract

**Background:**

Upper limb tendon/ligament injuries are common in sport and may require reconstruction using grafts or tendon transfers. Understanding tissue-specific properties is key to improving graft selection criteria and developing new interventions.

**Purpose:**

To characterize morphological, biomechanical, and biochemical properties of clinically important upper limb tendons, expanding available data on upper and lower limb tissues that are commonly injured or used as grafts.

**Study Design:**

Descriptive laboratory study.

**Methods:**

A total of 31 individual upper limb tendons were each retrieved from 8 fresh-frozen human cadavers (3 female, 5 male; aged 49-65 years): supraspinatus, infraspinatus, subscapularis, long head of biceps, distal biceps, distal triceps, brachioradialis, flexor carpi radialis, flexor carpi ulnaris, palmaris longus, extensor carpi radialis longus and brevis, extensor carpi ulnaris, flexor pollicis longus, abductor pollicis longus, extensor pollicis brevis and longus, extensor indicis proprius, extensor digiti minimi, and from the second to fifth digits, flexor digitorum superficialis and profundus and extensor digitorum communis. Outcome measures included tissue length; cross-sectional area, and major/minor axes; failure load; ultimate tensile strength (UTS); failure strain; elastic modulus; sulfated glycosaminoglycan (sGAG) and hydroxyproline content. Differences were analyzed using mixed linear regression models.

**Results:**

Rotator cuff tendons were distinct, exhibiting large, flat cross-sections with high failure loads and sGAG, and low UTS. Supraspinatus was significantly weaker with more sGAG than infraspinatus and subscapularis. Tendon UTS and elastic modulus were largely similar between elbow, wrist, and hand tendons. Exceptions included higher and lower material properties of the extensor pollicis brevis and flexor carpi ulnaris, respectively, than other distal upper limb tendons. Extensor tendons generally had higher sGAG than flexors with significant differences at the wrist and thumb, while hydroxyproline content was significantly higher in digital flexors versus extensors in the fingers.

**Conclusion:**

These findings enhance understanding of upper limb tendon properties, providing novel data to inform graft selection and tailor alternative engineered or regenerative strategies.

**Clinical Relevance:**

The combined upper/lower limb data sets include 48 unique appendicular tendons and ligaments sourced from the same donors and evaluated using consistent methodologies, providing a robust comparative database to aid selection of suitable donor tendons for the repair/reconstruction of injured tendons/ligaments and the development of graft materials.

Upper limb tendons and ligaments are essential for coordinated, dextrous movements but are susceptible to trauma (eg, digital tendon laceration or overuse tears). Sporting injuries may include acute distal biceps tendon rupture in eccentric contraction during weightlifting; digital flexor avulsions in rugby (jersey finger); ulnar collateral ligament of thumb avulsion in skiing, catching, or blocking ball sports (skiers’ or gamekeepers’ thumb); chronic overuse, impingement or acute traumatic tears of rotator cuff tendons; or attenuation or rupture of the medial ulnar collateral ligament of the elbow due to repeated overarm throwing actions.^15,37,38,45^ In the United States, >30% of all primary tendon ruptures occur in the upper limb, with approximately one-third of these cases linked to sports-related activities.^31^

High injury rates, combined with growing demand for rapid, full functional recovery and reduced reinjury risk, underscore the importance of improving knowledge of native tissue properties and refining surgical strategies to optimize patient outcomes.^18,31,35^ Although acute trauma in the upper limb is commonly treated through primary repair, reconstruction using tendon transfers or free tendon grafts is common for primary repair failure, delayed intervention, and ruptures associated with chronic degenerative disease (eg, tendinopathy, rheumatoid arthritis).^12,37,44,45^ In these instances, donor tendons from both upper and lower limbs have been utilized. Hand/wrist flexors and extensors are typically reconstructed using smaller tendon grafts (eg, palmaris longus [PL], plantaris, and extensor digitorum longus),^9^ while larger distal biceps tendons and elbow medial ulnar collateral ligaments have been reconstructed using PL, plantaris, gracilis, semitendinosus, and Achilles tendons.^19,36,41,42^ The long head of the biceps tendon (LHBT) has also been transposed to augment superior capsular reconstruction in irreparable rotator cuff tears, which are commonly reconstructed using dermal allografts or xenografts.^25,28^ Although patient-derived autografts optimize graft-host compatibility, allograft and xenograft use is increasing^14,25^ and necessary when autograft tissues are absent or inadequate (eg, PL and plantaris may be absent in up to 15%-20% of people^52,53^ and inadequately sized in >50% of cases^21^), previously used, or when several grafts are required for multi-tendon/multi-ligamentous injuries.

In the absence of large-scale clinical data comparing all grafts, detailed characterization of tissue properties of commonly injured tissues and potential graft sources is essential. This knowledge can help identify factors influencing injury susceptibility and inform graft selection or design based on mechanical and biological suitability. Previous studies of morphological and/or biomechanical properties have typically compared 2 to 5 graft types for upper limb reconstructions^9,21,42,49^ with only 1 study evaluating the biomechanics of 25 forearm, wrist, and hand tendons.^48^ Broader scale comparisons with other tendons of the elbow and shoulder, and lower limb graft options, remain limited; and biochemical properties are often absent. A comprehensive, multi-outcome assessment of variations present in different tendons and ligaments is critical for improving treatments designed to rapidly restore functional capacity across diverse locomotor activities.

The present study aimed to characterize the morphological, biomechanical, and biochemical properties of 31 human upper limb tendons (all are defined in Table 1), complementing previous lower limb research^2^ and expanding the musculoskeletal tissue database defining characteristics of normal human appendicular tendons and ligaments that are either commonly injured, reconstructed, or used/considered for grafts. All data generated and analyzed in the current study are openly available as data sets 1^3^ and 2^6^ at https://dataverse.harvard.edu/dataverse/human_musculoskeletal_tissue_datasets-tendon-ligament.

**Table 1:** Tendon Names and Abbreviations Split by Functional Region in Upper Limb

Tendons by Functional Region	Abbreviation
Shoulder	
Supraspinatus	SSP
Infraspinatus	IS
Subscapularis	SSC
Long head of biceps tendon	LHBT
Elbow	
Distal biceps	DBT
Brachioradialis	BrR
Distal triceps	DTT
Wrist	
Flexor carpi radialis	FCR
Palmaris longus	PL
Flexor carpi ulnaris	FCU
Extensor carpi radialis longus	ECRL
Extensor carpi radialis brevis	ECRB
Extensor carpi ulnaris	ECU
Fingers (2nd-5th digits)	
Flexor digitorum superficialis-2nd digit	FDS-2
Flexor digitorum superficialis-3rd digit	FDS-3
Flexor digitorum superficialis-4th digit	FDS-4
Flexor digitorum superficialis-5th digit	FDS-5
Flexor digitorum profundus-2nd digit	FDP-2
Flexor digitorum profundus-3rd digit	FDP-3
Flexor digitorum profundus-4th digit	FDP-4
Flexor digitorum profundus-5th digit	FDP-5
Extensor indicis proprius	EIP
Extensor digitorum communis-2nd digit	EDC-2
Extensor digitorum communis-3rd digit	EDC-3
Extensor digitorum communis-4th digit	EDC-4
Extensor digitorum communis-5th digit	EDC-5
Extensor digiti minimi	EDM
Thumb (1st digit)	
Flexor pollicis longus	FPL
Abductor pollicis longus	APL
Extensor pollicis brevis	EPB
Extensor pollicis longus	EPL

## Methods

### Tissue Acquisition

Fresh-frozen (–20°C) left and right upper limbs were sourced from 8 cadaveric donors with no known history of soft tissue musculoskeletal injuries or conditions (5 male, 3 female; aged 49-65 years) (see Supplemental Material 1, available separately; section A1.1).^2^ Informed consent was obtained by Science Care Inc from all donors or next of kin. Ethics approval was granted by the Northern Sydney Local Health District human research ethics committee.

### Tendons

One left or right limb per donor was randomly allocated to this study. A total of 31 anatomically distinct tendons were retrieved by the same surgeons (D.C., D.O.) (Table 1), wrapped in saline-soaked gauze, sealed, and stored at −20ºC. Supplemental Material 1 (available separately; section A1.2) contains a detailed dissection protocol including approach and proximal and distal end points of excised tissues. All subsequent steps were conducted as previously described^2^ with brief descriptions provided herein. Although no freeze-thaw cycling effects were expected,^5^ the number of cycles was consistent, occurring for tissue retrieval, morphological and mechanical testing, and biochemical assessment.

### Specimen Preparation

Tendons were thawed for 1 hour at room temperature before muscle, fat, and loose connective tissue were removed. Aponeuroses defined the relevant proximoal or distal boundary of the free tendon. The mechanical testing gauge length was marked by dyeing the free tendon midpoint and 2 equidistant lines set 50 mm apart, except for the shorter rotator cuff tendons (SSP, IS, and SSC), which had 15-mm gauge lengths. If the free tendon of an individual rotator cuff tendon was <15 mm, tissue beyond the aponeurosis boundary was included to reach the target length. Biomechanical data were excluded if failure occurred outside the free tendon region.

### Morphology Measurements

Tendon length was recorded with a ruler (1-mm increments). Transverse, cross-sectional ultrasound (US) images were captured by the same researcher (D.M.A.) at 10-mm intervals along the gauge length or as a single image at the midpoint for the rotator cuff tendons (Lumify L12-4 linear array transducer; Phillips Healthcare). As detailed in Supplemental Material 1 (available separately; section A1.3), the US-based cross-sectional area (CSA_US_), and major and minor axes of a fitted ellipse were measured using ImageJ 1.53t (National Institutes of Health). Only measurements at the center of the free tendon are presented.

### Biomechanical Testing

As previously reported,^1,2^ CSA was measured at the narrowest point within the gauge region using a contact-based protocol to calculate mechanical properties (CSA_M_). Specimens were gripped in dry ice cryogenic clamps and loaded to failure at 5% strain/s (Instron 8874; Instron Corp). Force and displacement were recorded at 100Hz (25kN loadcell; Instron Dynacell 2527-201; and Instron 8874 crosshead, respectively). Failure modes (“midsubstance,”“edge of clamps,”“within clamps,” or “grip slip”) were noted for reviewing data inclusions.

Failure load was calculated as the maximal measured force. The working gauge length, used to calculate strain, was defined at a 4-N threshold. Stress was calculated as force divided by CSA_M_, and stress-strain curves were generated to determine the ultimate tensile strength (UTS) and failure strain at the maximal measured force. Elastic modulus was defined as the maximal slope over a 2% strain range within the “linear” region.

### Biochemical Analysis

After mechanical testing, ~50 to 100 mg (wet tendon mass) was collected from the midregion and stored at −80°C. Adapted protocols^2^ were used to measure hydroxyproline and sulfated glycosaminoglycan (sGAG) contents in vacuum-dried, papain-digested tendon samples. Hydroxyproline, indicative of collagen content, was measured using the p-dimethylaminobenzaldehyde assay, with absorbance read at 562 nm and trans-4-Hydroxy-L-proline (Sigma-Aldrich) used as the standard. sGAG, representing proteoglycan content, was quantified using the 1,9-dimethylmethylene blue assay, with absorbance at 650 nm and bovine trachea chondroitin sulfate as the standard (Sigma-Aldrich).

### Statistical Analysis

An a priori power analysis (G*Power 3.1; HHU)^15^ determined that 190 samples were required to detect a moderate effect size (*f*^2^ = 0.15) with 80% power and *α* =.05. The current study included 246 tendons.

**Figure 1.**
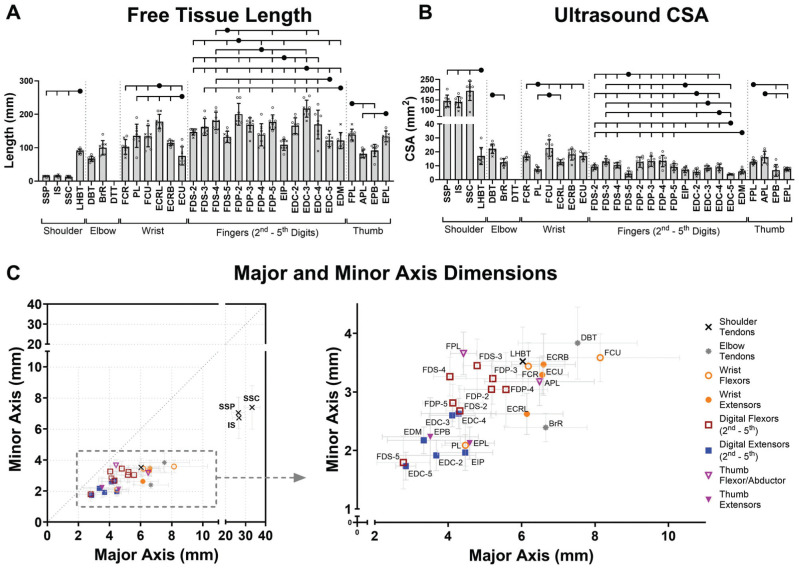
Morphological characterization of human tendons of the upper limb including (A) the free tissue length and (B, C) transverse ultrasound measures at the center of the free tissue. (A) Tissue length and (B) ultrasound cross-sectional area data are presented as mean ± SD alongside individual data points categorized by donor sex (x = female; o = male). Only significant differences between tendons within the same functional region are annotated (tick marks on the overhead brackets indicate a significant pairwise difference relative to the solid black circle). The complete set of significant pairwise differences is provided in Supplemental Material 2 (available separately). (C) Minor and major axis dimensions are presented as mean ± SD: all tissues and the box insert with scaled axes to distinguish the overlapping cluster of elbow, wrist, and hand tendons. All abbreviations are expanded in Table 1.

**Figure 2.**
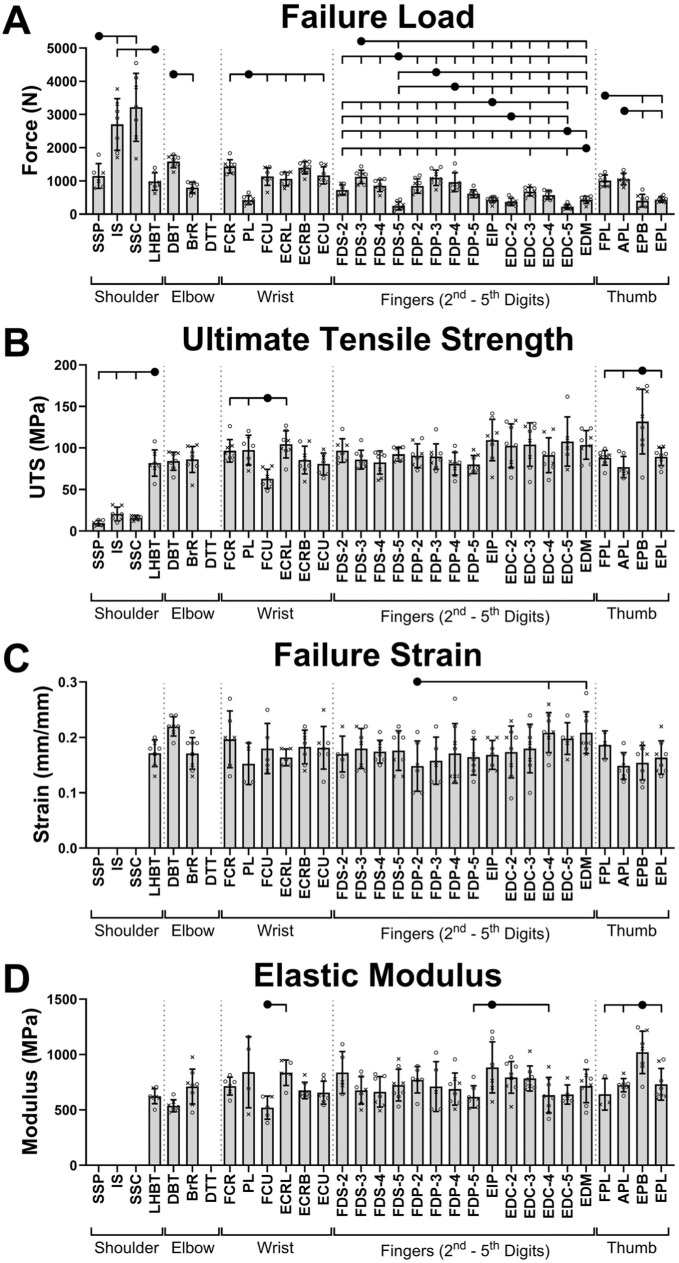
Biomechanical properties of human tendons of the upper limb: (A) failure load, (B) ultimate tensile strength (UTS), (C) failure strain, and (D) elastic modulus. Data are presented as mean ± SD alongside individual data points categorized by donor sex (x = female; o = male). Only significant differences between tendons within the same functional region are annotated (tick marks on the overhead brackets indicate a significant pairwise difference relative to the solid black circle). The complete set of significant pairwise differences is provided in Supplemental Material 2 (available separately). All abbreviations are expanded in Table 1.

**Figure 3.**
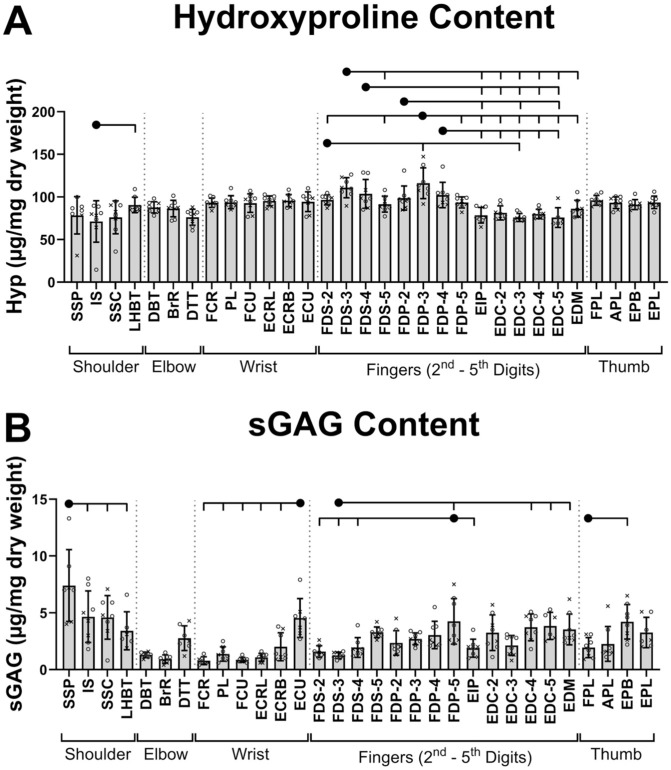
Biochemical properties of human tendons of the upper limb: (A) hydroxyproline (Hyp) content and (B) sulfated glycosaminoglycan (sGAG) content. Data are presented as mean ± SD with individual data points categorized by donor sex (x = female; o = male). Only significant differences between tendons within the same functional region are annotated (tick marks on the overhead brackets indicate a significant pairwise difference relative to the solid black circle). The complete set of significant pairwise differences is provided in Supplemental Material 2 (available separately). All abbreviations are expanded in Table 1.

Stata SE 15.1 (StataCorp) was used for all other analyses. Šidák-adjusted pairwise comparisons between all tendons were evaluated using mixed-effects linear regression models including donor age, sex, height, and weight as covariates and a random intercept term for donors. Following residual-based normality assessments, CSA and failure load outcomes were analyzed using generalized linear mixed-effects models with a gamma distribution and log link function, incorporating the same covariates and random-effects structure. Pearson correlation coefficients assessed associations between biomechanical and biochemical outcomes with adjustment for multiple comparisons using the Benjamini-Hochberg procedure (false discovery rate, 0.05). Statistical significance was set at adjusted *P* (adj*P*) values <.05. Only significant differences between tendons within the same functional region are highlighted in the Results and Figures 1 to 3. The complete set of comparisons across all tendons of the upper limb are in Supplemental Material 2 (available separately), which details the mean contrast (β, pairwise differences accounting for regression model covariates), adjusted and unadjusted *P* values, and 95% CIs.

Differences between flexor and extensor tendon groupings at various anatomical sites (wrist, fingers, thumb, knee, ankle, and toe) were evaluated using matched donor tendons from the upper (current study) and previously evaluated lower limbs^2,3^ (Supplemental Material 1, available separately; section A1.4). The dorsi- and plantarflexors of the ankle were coded as flexors and extensors, respectively. Šidák-adjusted, region-specific differences between the pooled flexors and extensors were identified using mixed linear regression models (covariates: age, sex, height and weight; random intercepts: donor and limb).

Supplemental Material 1 (available separately; section A1.6) details data exclusions, final n per tendon and outcome, and mean ± SD summaries. Rotator cuff tendons (SSC, IS, and SSC) were excluded from all strain-based analyses due to the shortened gauge length as discussed in previous research.^2^ Only the biochemical content of the DTT was analyzed due to the full-length integration of muscle and tendon tissues limiting intact tendon isolation for morphological and biomechanical assessments.

### Additional Data Set Outcomes

Additional morphological, biomechanical, and biochemical outcomes described in Supplemental Material 1 (available separately; section A1.5) are available at https://doi.org/10.7910/DVN/LXN3EH.^6^

## Results

### Morphology

Excluding rotator cuff tendons, the free tendon was on average 67% of the total length retrieved. In comparison, the free tendon comprised only 28% of the total rotator cuff tendon lengths. As a region (Figure 1A), the mean free tendon length of the rotator cuff tissues was 14 mm, while the LHBT averaged 89 mm and elbow tendons 83 mm. The wrist, fingers, and thumb had the longest free tendons, averaging 123 mm, 158 mm, and 112 mm, respectively.

The rotator cuff tendons had the greatest CSA_US_ (Figure 1B), averaging 159.89 mm^2^. These 3 tendons were significantly larger than all other upper limb tendons including the LHBT (adj*P* < .0001). The mean tendon CSA_US_ for the remaining regions was 17.39 mm^2^ for elbow, 15.70 mm^2^ for wrist, 8.96 mm^2^ for finger, and 10.79 mm^2^ for thumb. Within these regions, PL was significantly smaller than all other wrist tendons (adj*P*≤ .0005), FDS-5 and EDC-5 were significantly smaller than most digital flexors and some extensors in the hand (adj*P*≤ .009), and thumb flexors had significantly larger CSA than thumb extensors (adj*P*≤ .001).

The cross-sectional major and minor dimensions highlight the unique sheetlike appearance of the 3 rotator cuff tendons (Figure 1C). The remaining tendons in the data set had a more rounded cross-section with FPL and FDS-4 the most uniform, while DBT, BrR, and FCU had flatter cross-sections.

### Biomechanics

By region, the shoulder tendons had the highest mean failure load at 2045.14 N. As expected, the large IS and SSC had failure loads that were ≥139% and 183% greater than all other tendons, respectively (adj*P*≤ .0001). Interestingly, SSP failed at a significantly lower load than both IS (58% lower; adj*P* < .0001) and SSC (65% lower; adj*P* < .0001). Elbow and wrist tendons failed at similar loads, respectively, averaging 1166.17 N and 1115.12 N. Digital tendons had the lowest failure loads, aligning with their smaller CSA_US_ (finger tendons averaged 663.67 N; thumb tendons averaged 718.36 N). Within regions, there were significant differences between individual tendons (Figure 2A). The failure loads of the digital flexors were often significantly higher than the digital extensor tendons (fingers: ≥54% greater, adj*P*≤ .02; thumb: ≥230% greater, adj*P* < .0001).

After correcting for CSA, UTS was more similar between tendons (Figure 2B), particularly for elbow, wrist, finger, and thumb digital tendons, averaging 85.16 MPa, 88.37 MPa, 93.83 MPa, and 97.22 MPa, respectively. The mean regional UTS was lowest for shoulder tendons (32.83 MPa). Distinct tendons within regions included LHBT from the shoulder, which was significantly stronger than all rotator cuff tendons (|β| ≥ 60.79 MPa; *P* < .0001); FCU from the wrist being significantly weaker than FCR, PL, and ECRL tendons (|β| ≥ 33.58 MPa; *P*≤ .01); and the thumb EPB significantly stronger than all other thumb tendons (|β| ≥ 42.31 MPa; *P* < .0001).

Failure strain (Figure 2C) was not significantly different between most evaluated tendons (excluding rotator cuff tendons). Mean regional failure strains were 17.45% for LHBT at the shoulder, 19.41% for elbow, 17.82% for wrist, 17.82% for finger, and 16.03% for thumb. Only 7 tendons were significantly different from other tendons either within or across joint regions (see Supplemental Material 2, available separately). For example, FDP-2 and APL had significantly lower failure strains compared with EDC-4 and EDM (FDP-2: |β| ≥ 6.11%, adj*P*≤ .01; APL: |β| ≥ 5.94%, adj*P*≤ .009).

Elastic modulus (Figure 2D) was similarly not different between most tendons although region means varied: LHBT at the shoulder = 623.45 MPa, elbow = 629.59 MPa, wrist = 698.32 MPa, fingers = 723.52 MPa, and thumb = 798.88 MPa. Notable tendon differences within regions included the FCU being significantly lower than ECRL (|β|= 317.27 MPa; adj*P* = .03); while EIP was significantly higher than FDP-5 (|β| = 259.36 MPa; adj*P* = .04) and EDC-4 (|β| = 249.13 MPa; adj*P* = .04); and EPB was significantly higher than all other thumb tendons (|β| ≥ 279.81 MPa; adj*P*≤ .009).

### Biochemistry

Regionally, hydroxyproline (Figure 3A) averaged 79.1 µg/mg, 83.5 µg/mg, 94.2 µg/mg, 92.5 µg/mg, and 93.5 µg/mg for shoulder, elbow, wrist, finger, and thumb tendons, respectively. Uniquely, most finger flexor tendons had significantly higher hydroxyproline than finger extensors (|β| ≥ 20.1 µg/mg; adj*P*≤ .02).

The sGAG (Figure 3B) was more variable across tendons and regions (shoulder = 5.1 µg/mg, elbow = 1.7 µg/mg, wrist = 1.7 µg/mg, finger = 2.8 µg/mg, thumb = 2.9 µg/mg). Notable region-specific tendons with significantly elevated sGAG included the shoulder SSP (relative differences to other tendons within the same region: |β| ≥ 2.7 µg/mg; adj*P*≤ .002), wrist ECU (|β| ≥ 2.6 µg/mg; adj*P*≤ .008), FDP-5 in the fingers (|β| ≥ 2.3 µg/mg; adj*P*≤ .03), and thumb EPB (|β| = 2.3 µg/mg; adj*P* = .03).

### Associations

Table 2 summarizes correlations between biochemical and biomechanical outcomes. Across all tendons, CSA_US_ was negatively correlated with hydroxyproline (*r* = −0.31; adj*P* < .0001) while sGAG was positively correlated (*r* = 0.42; adj*P* < .0001). However, the direction of association changed when excluding the rotator cuff tendons. Similarly, failure load was only significantly correlated with hydroxyproline (*r* = 0.35; adj*P* < .0001) and sGAG (*r* = −0.36; adj*P* < .0001) after excluding rotator cuff tendons. Conversely, the negative association between sGAG and UTS (*r* = −0.32; adj*P* < .0001) was no longer significant when rotator cuff tendons were removed. No significant associations within the rotator cuff tendons were identified.

**Table 2:** Pearson Correlation Coefficients (*r*) and Adjusted *P* Values for Associations Between Outcomes After the Benjamini-Hochberg Correction*^a^*

	Hydroxyproline Content				sGAG Content			
Outcome	*r*	Adj*P*	*r^b^*	Adj*P^b^*	*r*	Adj*P*	*r^b^*	Adj*P^b^*
CSA_US_	**−0.31**	**<.0001**	**0.24**	**.002**	**0.42**	**<.0001**	**−0.24**	**.002**
Failure load	−0.07	.31	**0.35**	**<.0001**	0.08	.30	**−0.36**	**<.0001**
UTS	0.12	.11	**−0.19**	**.01**	**−0.32**	**<.0001**	0.04	.66
Failure strain			**−0.17**	**.04**			−0.09	.35
Elastic modulus			0.00	.99			0.04	.70
sGAG content	−0.11	.12	−0.07	.40				

a Correlations were evaluated for data sets including and excluding the unique rotator cuff tendons of the shoulder. Bold values indicate statistical significance after Benjamini-Hochberg adjustment (false discovery rate, 0.05). Strength of association: negligible (0.00 ≤ | *r* | < 0.30); weak (0.30 ≤ | *r* | < 0.50). Adj*P*, adjusted *P* value; CSA_US_, ultrasound-quantified cross-sectional area; sGAG, sulfated glycosaminoglycan; UTS, ultimate tensile strength.

b Rotator cuff tendons excluded.

### Regional Flexor-Extensor Differences

**Figure 4.**
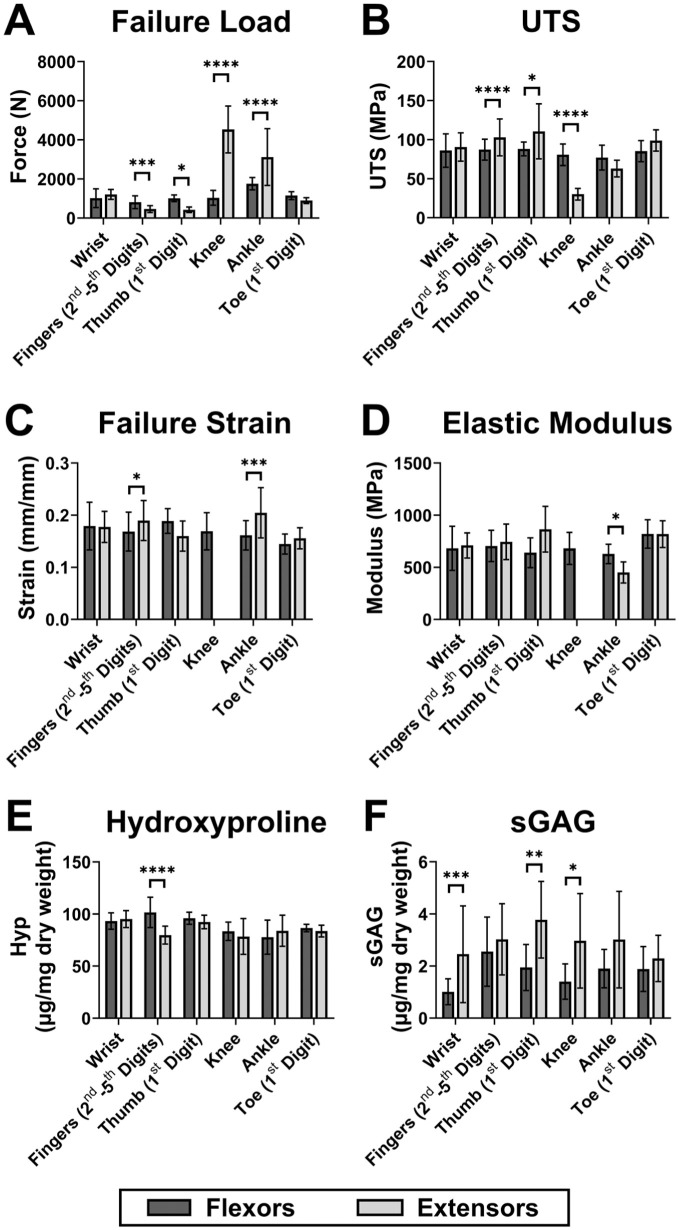
Flexor-extensor tendon properties across different anatomical sites in the upper and lower limbs with data presented as mean ± SD: (A) failure load, (B) ultimate tensile strength (UTS), (C) failure strain, (D) elastic modulus, (E) hydroxyproline (Hyp) content, and (F) sulfated glycosaminoglycan (sGAG) content. Šidák adjusted significant differences between flexors and extensors within the same functional region are annotated by asterisks (*adj *P* < .05; **adj *P* < .01; ***adj *P* < .001; ****adj *P* < .0001). Note that ankle flexors and extensors represent dorsiflexors and plantarflexors, respectively. The specific individual tendons utilized are detailed in Supplemental Material 1 (available separately; section A1.4).

Opposing tendon group differences were both region and outcome specific (Figure 4). FHL and EHL in the first toe were equivalent for all outcomes while wrist flexors and extensors only differed significantly in sGAG content (|β| = 1.5 µg/mg; adj*P* = .0005). Flexor tendons of the fingers and thumb exhibited significantly higher failure loads than their extensors (fingers |β| = 349.34 N, adj*P* = .0005; thumb |β| = 587.72 N, adj*P* = .02), while the lower limb knee extensors and ankle “extensors” (plantarflexors) reached significantly higher loads than their counterparts (knee |β| = 3492.32 N, adj*P* < .0001; ankle |β| = 1380.17 N, adj*P* < .0001). Conversely, when corrected for CSA, upper limb digital extensors were significantly stronger than digital flexors (UTS: fingers |β| = 15.50 MPa, adj*P* < .0001; thumb |β| = 22.48 MPa, adj*P* = .02), and knee flexors were significantly stronger than knee extensors (knee |β| = 49.57 MPa; adj*P* < .0001). Significant regional group differences for failure strain, elastic modulus, and hydroxyproline content were only observed in tendons of the fingers (failure strain |β| = 1.92%, adj*P* = .01; hydroxyproline |β| = 21.6 µg/mg, adj*P* < .0001) and ankle (failure strain |β| = 4.38%, adj*P* = .007; elastic modulus |β| = 187.65 MPa, adj*P* = .02). The mean sGAG content was consistently higher in extensor versus flexor tendons across all regions of the upper and lower limbs, although this was only significant for wrist, thumb, and knee tendons (|β| ≥ 1.5 µg/mg; adj*P*≤ .03).

## Discussion

Prior studies of upper limb tendon properties focused on select tendons within specific anatomical regions or on those most commonly used for autologous grafting because of their accessibility and low donor-site morbidity. However, advancements in surgical technique, improved tissue processing, and the increasing use of allografts support the need for a broader evaluation of potential graft options. This data set of upper limb tendon properties adds to previous work on lower limb tendons,^2,3^ providing an extensive, open-access database of appendicular tendon properties. The collection of data from the same donors using consistent methodologies crucially avoids the interstudy variability that typically arises from differences in donor characteristics, tissue processing, storage, and testing conditions. This comprehensive characterization of a broad spectrum of tendons offers valuable insights into the unique adaptations of specific tendons and tendon groups.

The rotator cuff tendons (SSP, IS, and SSC) are a key example of specialized tendons. Relative to other upper limb tendons, the shorter, sheetlike SSP, IS, and SSC exhibited substantially elevated failure loads and sGAG, characteristics that align with the large tensile, compressive, and shear forces that traverse through the highly mobile glenohumeral joint. Although all 3 rotator cuff tendons demonstrated significantly lower normalized tensile strengths than other tissues, this likely reflects their more heterogeneous fiber alignments, which accommodate multidirectional loading but are not wholly measured by uniaxial testing methods.^22,23^ The heterogeneous nature of the rotator cuff tendons and their higher mobility may also explain the greater rate of tears in these tendons compared with other tendons, particularly with increasing age.^44^

SSP is reportedly the most susceptible to traumatic and degenerative tears.^8^ Of the 3 rotator cuff tendons evaluated, SSP had the lowest maximal failure load, and although not significant, the lowest UTS. While the biomechanics of the rotator cuff tendons have not previously been compared at the whole-tendon level, the higher sGAG in SSP compared with other shoulder tendons has been previously reported.^4,39^ Given that elevated sGAG content has been associated with reduced tendon strength in both normal and pathological tissues,^2,10,39^ the unique biomechanical and biochemical characteristics of the SSP may contribute to its higher rate of injury.

The remaining tendons of the distal upper limb were largely distinct from the rotator cuff tendons. Tendon dimensions and maximal failure loads in the elbow, wrist, and hand still varied considerably; however, their material properties (UTS, failure strain, and elastic modulus) remained largely similar. This contrasts with the lower limb where both structural and material properties differed significantly across multiple tendons and ligaments,^2^ potentially reflecting greater functional diversity and loading variations among lower limb tendons (eg, positional control vs energy storage and power generation). Some notably distinct tendons in the distal upper limb included the wrist FCU, with the lowest UTS and elastic modulus; and the thumb EPB, which exhibited the highest UTS and modulus. Previous studies have not reported individual pairwise comparisons, but based on mean data, the FCU consistently has a lower UTS and elastic modulus than other wrist flexors and extensors.^29,48^ The lower strength and increased compliance may reflect adaptations associated with the FCU's significantly larger muscle mass and shorter fiber-to-muscle length ratios, which together help transfer and modulate greater muscle-generated forces without injury.^26,29^ In the EPB, however, Weber et al^48^ did not find the mean UTS or Young modulus of this tendon to be greater than other tendons of the forearm, wrist, and hand. It remains unclear whether this discrepancy stems from anatomical variations of this evolutionarily distinct tendon^43^ or differences in experimental methodology (eg, CSA estimations, strain rates, or modulus calculations),^48^ which invariably affect reported mechanical properties.^24^ From broader group comparisons, Weber et al similarly found that the UTS of digital extensors was significantly greater than that of digital flexors.^48^ However, they also reported a significantly higher Young modulus in digital extensors versus flexors and greater failure strain in wrist tendons compared with digital tendons. These conflicting results are likely due to specific tendons disproportionately influencing differences between groupings; for instance, the EDM Young modulus reported by Weber et al was more than double most other digital extensors, while FCR and ECRL drove higher failure strain differences in the wrist.^48^ Such discrepancies, even at higher group levels, underscore the challenges of consolidating and interpreting data from studies using distinct data sets and methodologies.

Biochemically, sGAG content was as varied in the upper limb as it was in the lower limb.^2^ From the higher level group comparisons, extensors generally contained more sGAG than flexors in both upper and lower limb tendons, although this was only significant in the wrist, thumb, and knee tendons (Figure 4F). In contrast, hydroxyproline was expected to be consistent across upper limb tendons based on lower limb findings^2^ and previous reports of the collagen content in human wrist flexors and extensors.^29^ However, digital flexor tendons demonstrated significantly elevated levels compared with the digital extensors, coinciding with significantly higher maximal failure loads (Figures 2A and 3A). This pattern was not observed in any other functional region and to the best of our knowledge, has not previously been reported. Two studies from the same group measuring collagen content in porcine digital flexor and extensor tendons support this trend.^50,51^ Conversely, equine superficial digital flexor and common digital extensor tendons demonstrated no collagen differences in thoroughbreds but elevated extensor collagen content in other breeds, while sGAG was substantially higher in all flexors versus extensors.^47^ Species- and tendon-specific loading environments and functional demands may drive these differences.

Potential tendon-specific factors were also apparent when exploring associations between biochemical and biomechanical tendon properties. Although significant associations between both hydroxyproline and sGAG and CSA_US_, as well as sGAG and UTS, were identified with all tendons combined, these were at most weakly correlated. The exclusion of the distinctive rotator cuff tendons from the pooled data set also altered the observed associations. This indicates that biochemical/biomechanical relationships are not uniform across all tendons and other factors may modulate these relationships, emphasizing the need for more tendon-specific characterization. The aggregate quantitation of hydroxyproline and sGAG content presents a further limitation, as these assays do not distinguish between different collagen types, GAG species, or cross-linking patterns that may be critical to understanding composition-function relationships. Donor age and sex may also moderate properties in a tendon-specific manner^1,20^; however, the limited number of only 8 individual donors precluded adequate adjustment for these potential covariates. Despite these limitations, the distinct clustering of the rotator cuff tendons highlights their unique compositional and functional adaptations, reinforcing the importance of characterizing distinct changes in different tendons/tendon groups in future studies.

Specialized tendon properties may have clinical significance for graft selection. While systematic reviews generally report reasonable patient outcomes irrespective of the graft selected, high-quality evidence from randomized controlled trials or large cohort studies remain limited for many graft options.^19,27,46^ In the absence of these, direct comparison of relevant tendon and ligament properties can provide some insight. Upper limb reconstructive surgeries utilize a range of donor tendons from both upper and lower limbs, with lower limb tendons typically providing greater tissue length.^2,30^ On the basis of mechanical properties, there is some flexibility in the selection of grafts because of the similarities between tendons of the upper limb although tissues with greater UTS may be preferred for allografts to offset any decreases in strength associated with different processing and sterilization protocols.^16^ Morphological aspects are also a concern when considering the intricate anatomy of the hand. Donor tendons used as free tendon grafts, or for tendon transfers, need to be of suitable length and cross-sectional dimensions when passing through sheaths, pulleys, and the retinacula. For digital flexor reconstruction, dimensional constraints are generally suitably addressed by the small PL tendon with favorable restoration of digital function reported in cadaveric studies.^52^ The plantaris provides an alternative when greater tissue length is required; however, the cross-sectional area is even smaller.^1,2,53^ While graft tensile strength (UTS) is often higher than the native digital flexor tendon,^2,34^ smaller cross-sectional areas with correspondingly lower failure loads present a greater risk of reinjury,^7^ particularly in sports with a high risk of digital overload (eg, rugby/football including flag- or tag-football).^38^

Compositional factors may also affect the performance of grafts postsurgery. sGAGs are naturally elevated in compressed tendon areas, particularly where they wrap around bony prominences, resulting in fibrocartilaginous regions to assist smooth articulation/gliding between surfaces.^4,17,32,33^ Adaptive changes to sGAG content in vivo has been investigated by translocating tensile and compressive regions of the rabbit flexor digitorum profundus.^17^ Transferring the compressed region to a more tensile-loading environment resulted in rapid loss of >60% sGAG, with its profile gradually resembling that of the native tensile regions. Interestingly, relocation to its original compressive setting resulted in minimal sGAG recovery. Similarly, repositioning a low-sGAG tensile tendon region into direct contact with the calcaneal bone did not alter total sGAG content. These results suggest that grafts with equivalent or elevated sGAG levels may be advantageous in accelerating the functional remodeling of implanted tissues when reconstructing tendons/ligaments with compressed regions (eg, digital flexors, rotator cuffs, and the medial ulnar collateral ligament). Regional variation in tendon composition and structure is also important to note as the biochemical analyses of the current study were restricted to a single midtendon region. The unknown degree of heterogeneity should be considered when relating findings to whole-tendon graft behavior and postimplantation remodeling. Additionally, as shown in other in vitro and in vivo studies, changes to external stresses such as stress deprivation^11,40^ and exercise^13,50,51^ can result in mechanical and biochemical adaptations in some tendons but not others, necessitating further research in this area to determine whether in vivo adaptations are graft specific, site specific, region specific, and/or time dependent.

Additional study limitations include the absence of upper limb ligaments; the inclusion of donors spanning a broader age spectrum, particularly younger donors; and the lack of dynamic, viscoelastic testing. These factors limit insight into age-related effects and time-dependent mechanical behaviors, and highlight important directions for future work. Furthermore, although donor limbs were randomized, limb dominance was not recorded and its potential impact could not be evaluated. Nevertheless, this study provides valuable insights into the quasistatic biomechanical properties, biochemical composition, and tissue-level morphology of upper limb tendons, providing a robust foundation for understanding tendon variability across different functional regions. Ongoing study of the histological and ultrastructural features of these distinct tissue types will deliver crucial insight into how tissue microarchitecture contributes to the observed morphological, biomechanical, and biochemical compositional differences.

## Conclusion

This comprehensive characterization of the morphological, biomechanical, and biochemical properties of upper limb tendons provides insights into the distinct characteristics of the sheetlike shoulder tendons; broad similarities in tendon strength and elastic moduli across tendons of the elbow, wrist, and hand, as well as surprisingly unique biochemical profiles in different functional groups. By establishing an extensive comparative database of musculoskeletal tissue properties using standardized methodology, this research builds on and consolidates existing knowledge, providing a critical reference point for advancing management of tendon injuries and degenerative conditions.

## Supplemental Material

sj-docx-1-ojs-10.1177_23259671261425673 – Supplemental material for The Biomechanical, Biochemical, and Morphological Properties of 31 Human Cadaveric Upper Limb Tendons: An Open Access Data Set Supplemental material, sj-docx-1-ojs-10.1177_23259671261425673 for The Biomechanical, Biochemical, and Morphological Properties of 31 Human Cadaveric Upper Limb Tendons: An Open Access Data Set by Carina L. Blaker, Dylan M. Ashton, David Chang, David O’Briain, Ying Liu, Samantha A. Hefferan, Nicholas Hartnell, Christopher B. Little and Elizabeth C. Clarke in Orthopaedic Journal of Sports Medicine

sj-xlsx-2-ojs-10.1177_23259671261425673 – Supplemental material for The Biomechanical, Biochemical, and Morphological Properties of 31 Human Cadaveric Upper Limb Tendons: An Open Access Data Set Supplemental material, sj-xlsx-2-ojs-10.1177_23259671261425673 for The Biomechanical, Biochemical, and Morphological Properties of 31 Human Cadaveric Upper Limb Tendons: An Open Access Data Set by Carina L. Blaker, Dylan M. Ashton, David Chang, David O’Briain, Ying Liu, Samantha A. Hefferan, Nicholas Hartnell, Christopher B. Little and Elizabeth C. Clarke in Orthopaedic Journal of Sports Medicine
