# Supplementary material for: The Biomechanical, Biochemical, and Morphological Properties of 31 Human Cadaveric Upper Limb Tendons: An Open Access Data Set
Source: Orthop J Sports Med. 2026 Apr 2;14(4):23259671261425673. doi: 10.1177/23259671261425673 (PMC13051129; doi:10.1177/23259671261425673)
Supplement: sj-docx-1-ojs-10.1177_23259671261425673 – Supplemental material for The Biomechanical, Biochemical, and Morphological Properties of 31 Human Cadaveric Upper Limb Tendons: An Open Access Data Set [file sj-docx-1-ojs-10.1177_23259671261425673.docx]

**Appendix A1**

**Title:** The biomechanical, biochemical and morphological properties of 31 human cadaveric upper limb tendons: an open access dataset part II

**Authors:** Carina L. Blaker^1,2,9^, Dylan M. Ashton^1,9^, David Chang^3^, David O’Briain^4,5,6^, Ying Liu^7^, Samantha A. Hefferan^1,7,9^, Nicholas Hartnell^8^, Christopher B. Little^7,9^, Elizabeth C. Clarke^1,9^^

1. Murray Maxwell Biomechanics Laboratory, Institute of Bone and Joint Research, Kolling Institute, Northern Sydney Local Health District, Faculty of Medicine and Health, University of Sydney, St. Leonards, New South Wales, Australia
2. Sydney School of Veterinary Science, Faculty of Science, The University of Sydney, Sydney, New South Wales, Australia
3. Department of Hand Surgery, Royal North Shore Hospital, Northern Sydney Local Health District, Sydney, New South Wales, Australia
4. University Hospital Waterford, Co Waterford, Ireland;
5. UPMC Whitfield, Waterford, Ireland;
6. University College Dublin School of Medicine, Dublin, Ireland
7. Raymond Purves Bone and Joint Research Laboratories, Institute of Bone and Joint Research, Kolling Institute, Northern Sydney Local Health District, Faculty of Medicine and Health, University of Sydney, St. Leonards, New South Wales, Australia
8. Bone Ligament and Tendon Pty Ltd, Bowral, New South Wales, Australia
9. The University of Sydney, Sydney Musculoskeletal Health, Australia

**^Corresponding author:**

Associate Professor Elizabeth Clarke

Murray Maxwell Biomechanics Laboratory,

Level 10, Kolling Institute B6,

The Royal North Shore Hospital,

St. Leonards, NSW 2065, Australia

Ph: +61-2-9926-4821

Fax: +61-2-9926-5266

Email: [elizabeth.clarke@sydney.edu.au](mailto:elizabeth.clarke@sydney.edu.au)

**Table of Contents**

[A1.1 Methods – Donor Characteristics 3](#_Toc203060202)

[Table A1. Donor information 3](#_Toc203060203)

[A1.2 Methods – Dissection Protocol 4](#_Toc203060204)

[A1.2.1 Specimen Retrieval 4](#_Toc203060205)

[A1.2.2 Anatomical variants/difficulties 6](#_Toc203060206)

[Table A2. List of tendon names and abbreviations split by functional region in the upper limb. 7](#_Toc203060207)

[A1.3 Methods – Ultrasound Analysis 8](#_Toc203060208)

[A1.4 Methods – Flexor-Extensor Tendon Groups 8](#_Toc203060209)

[Table A3: Individual tendons used for the analysis of flexor-extensor tendon groups. 8](#_Toc203060210)

[A1.5 Results – Additional Outcomes Included in the Online Dataset 9](#_Toc203060211)

[A1.6 Results - Sample Exclusions 10](#_Toc203060212)

[A1.7 Results – Data Summaries 10](#_Toc203060213)

[Table A4. Morphological and biochemical properties of human arm tendons. 11](#_Toc203060214)

[Table A5. Biomechanical properties of human arm tendons. . 12](#_Toc203060215)

[A1.8 Results – Pairwise Comparisons 13](#_Toc203060216)

[Table A6. Statistical summary from mixed-effects linear regression analysis*.* 13](#_Toc203060217)

[A1.9 Appendix References 25](#_Toc203060218)

Methods – Donor Characteristics

Table A1. Donor information

| **Donor ID** | **Sex** | **Age** | **Height in cm (in)** | **Weight in kg (lbs)** | **BMI** |
| --- | --- | --- | --- | --- | --- |
| D1 | M | 50 | 165 (65) | 60 (132) | 21.96 |
| D2 | F | 57 | 168 (66) | 50 (110) | 17.75 |
| D3 | M | 51 | 168 (66) | 54 (120) | 19.37 |
| D4 | M | 57 | 180 (71) | 59 (130) | 18.13 |
| D5 | F | 49 | 163 (64) | 65 (143) | 24.54 |
| D6 | F | 62 | 163 (64) | 54 (120) | 20.6 |
| D7 | M | 58 | 168 (66) | 59 (130) | 20.98 |
| D8 | M | 65 | 183 (72) | 53 (117) | 15.87 |
| Causes of death include: cardiopulmonary arrest (n=5); metastatic rectal cancer (n=1); liver failure (n=1); cerebral vascular disease (n=1) | | | | | |

Methods – Dissection Protocol

- 1. Specimen Retrieval

Upper limbs including the scapula through to the finger-tips were partially thawed at room temperature for 24 hours and transected at the level of mid-humerus to separate the distal aspect of the arm from the shoulder. The proximal humerus and shoulder were then re-wrapped and frozen at -20°C while the distal arm continued to be thawed at room temperature for a total of 36 hours. The dissection of the proximal shoulder specimens were completed after a second 24 hour thaw at room temperature. Thirty-one anatomically distinct tendons were retrieved from each limb (Table A2). All distal and proximal specimens were retrieved by the same surgeons in the same sequence to facilitate accurate identification, labelling and safe removal. Distal specimens from the mid-humerus to finger-tip were retrieved by a surgical fellow (DC) while the proximal shoulder specimens were retrieved by an orthopaedic surgeon (DO’B). One left or right limb per donor was randomly allocated to the outcomes included in this study. Any specimens which were damaged during retrieval were replaced by the same tendon in the contralateral limb. Following removal, all tissues were wrapped in saline-soaked gauze, sealed in double zip-locked plastic bags, and stored at -20ºC. All specimens were labelled with donor number, limb side, and tissue name.

*Distal specimens (mid-humerus to finger-tip)*

Each tendon was identified and released distally then followed proximally and released at the musculotendinous junction. Pronator teres, pronator quadratus, supinator and anconeus were not harvested as they do not have tendon segments long enough for evaluation in this study.

The flexor surface began with a transverse incision over the flexor retinaculum, and a longitudinal incision over the carpal tunnel, extending proximally in line with palmaris longus (PL). PL, flexor carpi radialis (FCR) and flexor carpi ulnaris (FCU) were sampled first, as the most superficial and easily identifiable structures. PL was released distally as it inserted into palmar fascia. FCR was incised distally as it passed underneath the tubercle of the trapezium. FCU was incised distally at its insertion into pisiform, as tendon containing this sesamoid bone was not useable in this study. The carpal tunnel was incised, allowing identification of flexor pollicis longus (FPL) to the thumb, and a flexor digitorum superficial (FDS) and flexor digitorum profundus (FDP) to the second/index, third/middle, fourth/ring and fifth/little digits (identified respectively as -2, -3, -4, -5). These tendons were followed, incising the overlying skin, fat and fascia, taking great care when releasing the flexor sheath and pulley system to each finger and thumb. Multiple vincula, nutrient supplying bands of connective tissue binding flexor tendons to volar surfaces of phalanx bones, were incised. Each FDS, FDP and FPL was incised at its bony insertion to maximise tendon length.

Each extensor surface began with a transverse incision over the extensor retinaculum of the wrist, with a longitudinal incision along the course of extensor digitorum to the middle finger. Tendons were excised by their extensor compartments. The first extensor compartment was released, identifying abductor pollicis longus (APL) and extensor pollicis brevis (EPB). The third extensor compartment was released, identifying extensor pollicis longus (EPL). Each of APL, EPB and EPL were released at their bony attachments. The second compartment, now clear of the previous three tendons, was released identifying extensor carpi radialis brevis (ECRB) and longus (ECRL). These were again released at their respective bony insertions. The fourth compartment was opened, revealing extensor indicis proprius (EIP) and the extensor digitorum communis (EDC) to the second (-2), third (-3), fourth (-4) and fifth (-5) digits. The fifth compartment was also released, revealing extensor digiti minimi (EDM). EDC contains a bulk of extensor hood and tendon together overlying the metacarpophalangeal (MCP) joint. As the extensor tendon distal to this bulky area splits and reforms, the tendon distal to this point was deemed not useful for the purposes of this study, hence EDC was incised distally at the level of the MCP joint. EIP and EDM were incised at the same level of EDC, as each respective tendon combined with EDC at this level. The sixth compartment was released, revealing extensor carpi ulnaris (ECU), which was incised at its bony origin at the base of the little finger metacarpal. Brachioradialis (BrR) was harvested after all other long tendons were removed, itself being removed at its broad insertion into the distal radius.

The common distal biceps (DBT) and triceps (DTT) tendons were harvested last. As the specimens began at the level of mid-humerus, DBT and DTT were identified in their muscle belly and released from surrounding tissue heading distally to their insertion. DBT was incised at its bony insertion into the radial tuberosity, while DTT was incised through its broad olecranon insertion.

*Proximal specimens (shoulder)*

The retrieval of the tendons of the shoulder began with an incision from the midpoint of the acromion, in line with the humeral shaft inferiorly, and extended superiorly over the trapezius. Full thickness skin flaps were elevated anteriorly and posteriorly. The deltoid muscle origin was then released from the scapular spine, acromion and lateral clavicle. The deltoid muscle was reflected inferiorly on its insertion to gain access to the underlying muscle layer. The conjoint tendon was released from the coracoid process and reflected inferiorly to gain access to the subscapular fossa. The trapezius insertion was then released from the scapular spine, acromion and lateral clavicle, and reflected proximally to gain access to the suprascapular fossa.

The long head of biceps tendon (LHBT) was identified within the bicipital groove of the humerus and the transverse humeral ligament released to mobilise the LHBT. The pectoralis major muscle was released from the lateral lip of the bicipital groove to expose the underlying biceps muscle. The convergence of the long and short head of biceps was identified inferiorly and the LHBT released at this level. Proximally, the LHBT was released from the supraglenoid tubercle. To retrieve the subscapularis (SSC) tendon, the superior border was released from the rotator interval and adhesions from the coracoid were also released. The inferior plane was developed between the subscapularis and latissimus dorsi. The muscle was divided medially and proximally in the middle subscapular fossa of the scapula. The SSC tendon was then peeled off from the lesser tuberosity insertion site. Inferiorly, the plane between the infraspinatus and teres minor was developed and continued laterally to the greater tuberosity to access the infraspinatus (IS) tendon. The IS was released from the scapular spine and fossa. Superiorly, the scapular spine defined the superior border with the supraspinatus and this separation was developed laterally to the greater tuberosity. Proximo-medially, the muscle was divided in the middle of the infraspinatus fossa. The IS tendon was then peeled off from the greater tuberosity insertion site. The posterior border of the supraspinatus (SSP) tendon was defined by the scapular spine and the infraspinatus. This posterior border was developed laterally to the greater tuberosity. The anterior border was released from the rotator interval. Proximo-medially, the muscle was divided in the middle of the supraspinous fossa. The SSP tendon was then peeled off from the greater tuberosity insertion site.

- 1. Anatomical variants/difficulties
- Palmaris longus is reported to be absent in approximately 15% of the total population, however this figure varies greatly depending on ethnic group.^5^ This tendon was present in all 8 donors.
- The flexor digitorum superficialis in the 5^th^ digit (FDS-5) is absent in approximately 6% of people.^9^ While the size varied greatly in all specimens, it was not absent from any donor in this study.
- Extensor digitorum communis (EDC) tendons were often linked with juncturae tendinum and were present in highly varied configurations as previously reported.^10^ The juncturae tendinum structures were trimmed from the sides of each EDC tendon to not affect subsequent analyses. Additionally, the EDC to the 5th digit (EDC-5) is reported to be absent in approximately 60% of cases^10^. In the present study, EDC-5 was absent from both left and right limbs of two male donors.
- For the outcomes included in this study, the distal triceps tendon (DTT) was only collected for biochemical measures due to the close integration and adherence of muscle fibres along the full length of tendon,^6^ hindering a clean dissection of the tendon proper without potentially damaging the tissue prior to morphological assessment and mechanical testing.

Table A2. List of tendon names and abbreviations split by functional region in the upper limb.

| **Tendons by Functional Region** | | **Abbreviation** |  |
| --- | --- | --- | --- |
| *Shoulder* | |  |  |
|  | Supraspinatus | SSP |  |
|  | Infraspinatus | IS |  |
|  | Subscapularis | SSC |  |
|  | Long head of biceps tendon | LHBT |  |
| *Elbow* | |  |  |
|  | Distal biceps | DBT |  |
|  | Brachioradialis | BrR |  |
|  | Distal triceps | DTT |  |
| *Wrist* | |  |  |
|  | Flexor carpi radialis | FCR |  |
|  | Palmaris longus | PL |  |
|  | Flexor carpi ulnaris | FCU |  |
|  | Extensor carpi radialis longus | ECRL |  |
|  | Extensor carpi radialis brevis | ECRB |  |
|  | Extensor carpi ulnaris | ECU |  |
| *Fingers (2^nd^ – 5^th^ Digits)* | |  |  |
|  | Flexor digitorum superficialis-2^nd^ digit | FDS-2 |  |
|  | Flexor digitorum superficialis-3^rd^ digit | FDS-3 |  |
|  | Flexor digitorum superficialis-4^th^ digit | FDS-4 |  |
|  | Flexor digitorum superficialis-5^th^ digit | FDS-5 |  |
|  | | Flexor digitorum profundus-2^nd^ digit | FDP-2 |
|  | Flexor digitorum profundus-3^rd^ digit | FDP-3 |  |
|  | Flexor digitorum profundus-4^th^ digit | FDP-4 |  |
|  | Flexor digitorum profundus-5^th^ digit | FDP-5 |  |
|  | Extensor indicis proprius | EIP |  |
|  | Extensor digitorum communis-2^nd^ digit | EDC-2 |  |
|  | Extensor digitorum communis-3^rd^ digit | EDC-3 |  |
|  | Extensor digitorum communis-4^th^ digit | EDC-4 |  |
|  | Extensor digitorum communis-5^th^ digit | EDC-5 |  |
|  | Extensor digiti minimi | EDM |  |
| *Thumb (1^st^ Digit)* | |  |  |
|  | Flexor pollicis longus | FPL |  |
|  | Abductor pollicis longus | APL |  |
|  | Extensor pollicis brevis | EPB |  |
|  | Extensor pollicis longus | EPL |  |

Methods – Ultrasound Analysis

Tendons were submerged in a saline bath before imaging with the Lumify L12-4 transducer using the following image acquisition settings: mechanical index 0.9, frame rate 24 Hz, gain 54, depth 35 mm, power -0.3 dB. The collected images were imported into ImageJ for analysis. Tissue borders were delineated using a default minimum threshold of 80. A binary mask was then generated to define the region of interest (ROI) which was restricted to areas with a pixel intensity > 0.8 and circularity > 0.4. Where the mask overlay did not correctly encompass the tendon cross-section, the threshold and circularity parameters were manually adjusted until an appropriate ROI was identified (ranges: threshold 60-110, circularity 0.1-0.4). The ellipse used for measuring the major and minor axes was fitted using the least squares method with respect to area. Measurements were calibrated using the scalebar included in each ultrasound image.

Methods – Flexor-Extensor Tendon Groups

Table A3: Individual tendons used for the analysis of flexor-extensor tendon groups across different functional regions of the upper and lower limbs.

| **Functional Region** | | **Flexion (*Dorsiflexion)** | **Extension (^#^Plantar Flexion)** |
| --- | --- | --- | --- |
| *Wrist* | | FCR | ECRL |
|  |  | PL | ECRB |
|  |  | FCU | ECU |
| *Fingers (2^nd^ – 5^th^ Digits)* | | FDS-2, -3, -4, -5 | EDC-2, -3, -4, -5 |
|  |  | FDP-2, -3, -4, -5 | EIP |
|  |  |  | EDM |
| *Thumb (1^st^ Digit)* | | FPL | EPB |
|  |  |  | EPL |
| *Knee* | | ST | QT |
|  |  | GT | PT |
| *Ankle* | | TA* | AT^#^ |
|  |  |  | TP^#^ |
| *Toe (1^st^ Digit/Hallux)* | | FHL | EHL |
| *Key: FCR, flexor carpi radialis; PL, palmaris longus, FCU; flexor carpi ulnaris; ECRL, extensor carpi radialis longus; ECRB, extensor carpi radialis brevis; ECU, extensor carpi ulnaris; FDS, flexor digitorum superficialis; FDP, flexor digitorum profundus; EDC, extensor digitorum communis; EIP, extensor indicis proprius; EDM, extensor digitorum minimi; FPL, flexor pollicis longus; EPB, extensor pollicis brevis; EPL, extensor pollicis longus; ST, semitendinosus tendon; GT, gastrocnemius tendon; QT, quadriceps tendon; PT, patellar tendon; TA, tibialis anterior; AT, Achilles tendon; TP, tibialis posterior; FHL, flexor hallucis longus; EHL, extensor hallucis longus.* | | | |

Results – Additional Outcomes Included in the Online Dataset

As previously reported for tendons and ligaments from the lower limb,^2^ data for the following additional outcomes are included in the full dataset available at <https://doi.org/10.7910/DVN/LXN3EH>.^4^

**Morphology**: total length of the excised specimen in addition to the free tendon length; and ultrasound-based CSA_US_ and minor and major axes’ dimensions from the image with the smallest CSA as an alternative non-contact CSA measurement for potential use in biomechanical analyses.

**Biomechanics**: multiple objective methods of calculating the elastic modulus to increase data usability: (i) the elastic modulus calculated at a defined strain (4%, 6%, 8% and 10%) from linear regression fit over a 2% strain range (i.e. 3-5%, 5-7%, 7-9% and 9-11% strain respectively); and (ii) the elastic modulus defined by the maximum gradient of a fifth-order polynomial fitted to the stress-strain data between 0% strain and strain at failure, confirmed within the “linear” region, as previously reported.^1,2^

**Biochemistry**: dry tissue weight and the conversion of hydroxyproline content to an approximation of total collagen content using a factor of 7.4, assuming collagen contains 13.5% hydroxyproline and the elastin content is negligible.^8^ This follows common practice in the field but we note that elastin, which is ~2-4% hydroxyproline,^3^ is actually variably present across different tendons and ligaments and will be separately quantified in future studies.^7^

Results - Sample Exclusions

**Retrieval:** all but two tendon specimens were successfully retrieved (n=246), with EDC-5 absent from two male donors. All DTT specimens were excluded from morphological and biomechanical assessment as the unique anatomy of this tendon prevented adequate discrimination and intact separation of the tendon proper from its integrated muscle tissue. Only biochemical assessment of the DTT was performed due to the smaller sampling requirements.

**Morphology:** ultrasound data (CSA, major and minor axes) could not be determined for two specimens due to missing images. Additionally, the major and minor axes were not determined for 2 EDM specimens due to noted bifurcations in the imaged region.

**Biomechanics:** ten specimens slipped, or failed within the clamps, and were excluded from the analysis of mechanical properties (failure load, UTS, failure strain, elastic modulus). One additional specimen was excluded from the analysis of mechanical properties as pre-existing tendon damage was identified at the time of testing. In line with our previous work,^2^ the shorter rotator cuff tendons (SSC, IS, SSP) were excluded from strain-based analyses (failure strain and elastic modulus). The reduced gauge length and distance between the cryoclamps (15 mm) increases the cooling effect and the potential for artificial tissue rigidity to develop within the gauge region if tissue freezing extends beyond the clamps. Thus, strain-based outcomes were conservatively dropped for this subset of tissues, and failure load and UTS reported only when the failure occurred in a non-frozen region. Of the remaining tendons, all specimens with an initial load below the ‘slack threshold’ of 4 N were included in strain-based analyses. There were 31 specimens with an initial load greater than 4 N. On review, 25 were excluded from strain-based analyses as the initial load was above 20 N and/or greater than 1% of the failure load. The data was retained for six specimens as the initial loads (ranging from 4.32 to 11.26 N) were less than 1% of their respective failure loads (ranging from 0.46% to 0.96%).

**Biochemistry:** nine samples were excluded from the analysis of sGAG content due to background interference (measured as high blank readings; absorbance > 0.1). One sample was excluded from hydroxyproline content analyses as it was outside the range of the standard curve.

Results – Data Summaries

The following tables provide the statistical summary of the measured data across all outcomes reported in the manuscript.

Table A4. Morphological and biochemical properties of human arm tendons. Reported as mean ± standard deviation. Free length refers to the tendon length up to the aponeurosis. The elliptical minor and major axes, and cross-sectional area (CSAUS) were measured from ultrasound images.

|  |  |  | **Morphology** | | | | |  |  | **Biochemistry** | | | |
| --- | --- | --- | --- | --- | --- | --- | --- | --- | --- | --- | --- | --- | --- |
| **Tissue** | **N** | **Free Length (mm)** |  | **N** | **Minor (mm)** | **Major (mm)** | **CSA_US_ (mm^2^)** |  | **N** | **Hyp (µg/mg)** |  | **N** | **sGAG (µg/mg)** |
| SSP | 8 | 14 ± 2 |  | 8 | 7.04 ± 1.01 | 26.32 ± 3.37 | 145.46 ± 28.37 |  | 7 | 78.5 ± 21.9 |  | 7 | 7.4 ± 3.2 |
| IS | 8 | 15 ± 3 |  | 8 | 6.72 ± 1.34 | 26.65 ± 3.38 | 139.57 ± 26.75 |  | 8 | 71.2 ± 24.3 |  | 8 | 4.7 ± 2.3 |
| SSC | 8 | 13 ± 3 |  | 8 | 7.39 ± 1.40 | 33.21 ± 4.00 | 194.64 ± 46.91 |  | 8 | 76.1 ± 19.4 |  | 8 | 4.6 ± 1.9 |
| LHBT | 8 | 89 ± 7 |  | 8 | 3.52 ± 0.59 | 6.03 ± 1.15 | 17.03 ± 6.15 |  | 8 | 90.6 ± 9.0 |  | 6 | 3.4 ± 1.7 |
| DBT | 8 | 67 ± 8 |  | 8 | 3.83 ± 0.61 | 7.53 ± 1.62 | 22.23 ± 3.45 |  | 8 | 87.8 ± 6.6 |  | 7 | 1.3 ± 0.2 |
| BrR | 8 | 100 ± 22 |  | 8 | 2.39 ± 0.24 | 6.66 ± 1.13 | 12.54 ± 2.62 |  | 8 | 86.4 ± 9.7 |  | 7 | 0.9 ± 0.4 |
| DTT | 0 | – |  | 0 | – | – | – |  | 8 | 76.2 ± 9.4 |  | 7 | 2.8 ± 1.1 |
| FCR | 8 | 103 ± 22 |  | 8 | 3.44 ± 0.40 | 6.18 ± 1.00 | 16.52 ± 2.09 |  | 8 | 93.3 ± 5.5 |  | 7 | 0.8 ± 0.4 |
| PL | 8 | 136 ± 35 |  | 8 | 2.09 ± 0.36 | 4.47 ± 0.64 | 7.33 ± 1.67 |  | 8 | 93.8 ± 7.6 |  | 8 | 1.4 ± 0.6 |
| FCU | 8 | 134 ± 32 |  | 8 | 3.58 ± 0.41 | 8.14 ± 2.16 | 22.76 ± 5.89 |  | 8 | 92.8 ± 10.8 |  | 8 | 0.9 ± 0.2 |
| ECRL | 8 | 178 ± 22 |  | 8 | 2.62 ± 0.35 | 6.14 ± 0.98 | 12.53 ± 1.80 |  | 8 | 95.3 ± 5.7 |  | 8 | 1.1 ± 0.4 |
| ECRB | 8 | 114 ± 8 |  | 8 | 3.47 ± 0.52 | 6.60 ± 0.87 | 18.09 ± 4.00 |  | 8 | 95.7 ± 7.0 |  | 8 | 2.0 ± 1.2 |
| ECU | 8 | 76 ± 27 |  | 8 | 3.29 ± 0.32 | 6.56 ± 0.71 | 16.95 ± 2.31 |  | 8 | 94.4 ± 11.5 |  | 7 | 4.5 ± 1.7 |
| FDS-2 | 8 | 147 ± 9 |  | 8 | 2.68 ± 0.43 | 4.32 ± 0.38 | 9.07 ± 1.57 |  | 8 | 96.6 ± 5.9 |  | 8 | 1.6 ± 0.5 |
| FDS-3 | 8 | 162 ± 25 |  | 7 | 3.45 ± 0.46 | 4.79 ± 0.39 | 12.98 ± 2.09 |  | 8 | 110.8 ± 11.7 |  | 8 | 1.3 ± 0.3 |
| FDS-4 | 8 | 180 ± 26 |  | 8 | 3.26 ± 0.33 | 4.05 ± 0.45 | 10.42 ± 1.89 |  | 8 | 103.6 ± 16.8 |  | 8 | 2.0 ± 0.9 |
| FDS-5 | 8 | 133 ± 17 |  | 8 | 1.80 ± 0.45 | 2.78 ± 0.72 | 4.12 ± 2.08 |  | 8 | 91.6 ± 9.4 |  | 8 | 3.3 ± 0.5 |
| FDP-2 | 8 | 200 ± 33 |  | 8 | 3.05 ± 0.49 | 5.18 ± 0.90 | 12.59 ± 3.65 |  | 8 | 98.7 ± 14.1 |  | 8 | 2.3 ± 1.1 |
| FDP-3 | 8 | 168 ± 21 |  | 8 | 3.23 ± 0.44 | 5.22 ± 1.21 | 13.24 ± 3.66 |  | 8 | 116.1 ± 18.1 |  | 8 | 2.7 ± 0.5 |
| FDP-4 | 8 | 139 ± 33 |  | 8 | 3.04 ± 0.63 | 5.57 ± 1.13 | 13.40 ± 4.11 |  | 8 | 102.3 ± 14.8 |  | 8 | 3.0 ± 1.2 |
| FDP-5 | 8 | 176 ± 22 |  | 8 | 2.81 ± 0.32 | 4.13 ± 0.82 | 9.21 ± 2.43 |  | 8 | 93.5 ± 6.5 |  | 8 | 4.2 ± 2.0 |
| EIP | 8 | 109 ± 16 |  | 8 | 1.96 ± 0.30 | 4.47 ± 0.75 | 7.00 ± 1.99 |  | 8 | 78.6 ± 9.1 |  | 8 | 1.9 ± 0.8 |
| EDC-2 | 8 | 166 ± 24 |  | 8 | 1.92 ± 0.26 | 3.68 ± 1.08 | 5.63 ± 1.99 |  | 8 | 81.4 ± 8.2 |  | 8 | 3.3 ± 1.6 |
| EDC-3 | 8 | 217 ± 25 |  | 8 | 2.60 ± 0.20 | 4.11 ± 0.63 | 8.41 ± 1.57 |  | 8 | 75.8 ± 4.7 |  | 8 | 2.1 ± 0.9 |
| EDC-4 | 8 | 169 ± 43 |  | 7 | 2.63 ± 0.26 | 4.29 ± 1.02 | 8.87 ± 2.27 |  | 8 | 80.0 ± 5.5 |  | 8 | 3.7 ± 1.2 |
| EDC-5 | 6 | 122 ± 18 |  | 6 | 1.73 ± 0.24 | 2.85 ± 0.27 | 3.85 ± 0.41 |  | 6 | 75.8 ± 11.5 |  | 6 | 3.8 ± 1.2 |
| EDM | 8 | 121 ± 24 |  | 8 | 2.17 ± 0.23 | 3.34 ± 0.99 | 5.86 ± 1.59 |  | 8 | 86.2 ± 9.9 |  | 8 | 3.5 ± 1.4 |
| FPL | 8 | 139 ± 16 |  | 8 | 3.66 ± 0.36 | 4.42 ± 0.35 | 12.68 ± 1.61 |  | 8 | 96.0 ± 5.8 |  | 8 | 1.9 ± 0.9 |
| APL | 8 | 82 ± 12 |  | 8 | 3.18 ± 0.41 | 6.49 ± 1.49 | 16.21 ± 4.19 |  | 8 | 93.3 ± 6.9 |  | 8 | 2.3 ± 1.5 |
| EPB | 8 | 92 ± 18 |  | 8 | 2.23 ± 0.67 | 3.51 ± 1.31 | 6.67 ± 4.22 |  | 8 | 91.2 ± 5.8 |  | 8 | 4.2 ± 1.5 |
| EPL | 8 | 134 ± 16 |  | 8 | 2.13 ± 0.27 | 4.59 ± 0.67 | 7.59 ± 1.05 |  | 8 | 93.6 ± 7.2 |  | 7 | 3.3 ± 1.3 |

Table A5. Biomechanical properties of human arm tendons as determined from tensile loading to failure. Reported as mean ± standard deviation.

|  | **Biomechanical Properties** | | | | | | | | | | | |
| --- | --- | --- | --- | --- | --- | --- | --- | --- | --- | --- | --- | --- |
| **Tissue** | **Target Gauge (mm)** |  | **N** | **CSA_M_ (mm^2^)** |  | **N** | **Failure Load (N)** | **UTS (MPa)** |  | **N** | **Failure Strain (%)** | **Elastic Modulus (MPa)** |
| SSP | 15 |  | 8 | 134.98 ± 33.69 |  | 6 | 1149.34 ± 373.08 | 9.54 ± 3.29 |  | 0 | – | – |
| IS | 15 |  | 8 | 133.35 ± 26.15 |  | 7 | 2700.13 ± 777.07 | 20.53 ± 8.22 |  | 0 | – | – |
| SSC | 15 |  | 8 | 212.12 ± 67.64 |  | 7 | 3218.56 ± 1022.97 | 16.16 ± 2.51 |  | 0 | – | – |
| LHBT | 15 |  | 8 | 13.73 ± 5.13 |  | 7 | 984.57 ± 262.59 | 81.75 ± 15.76 |  | 7 | 17.45 ± 2.47 | 623.45 ± 67.89 |
| DBT | 50 |  | 8 | 19.08 ± 3.35 |  | 7 | 1584.40 ± 186.61 | 84.01 ± 10.98 |  | 7 | 22.08 ± 1.70 | 536.80 ± 55.44 |
| BrR | 50 |  | 8 | 9.55 ± 2.39 |  | 8 | 800.22 ± 160.49 | 86.16 ± 15.68 |  | 8 | 17.08 ± 2.78 | 710.79 ± 157.06 |
| DTT | – |  | 0 | – |  | 0 | – | – |  | 0 | – | – |
| FCR | 50 |  | 8 | 14.89 ± 1.20 |  | 8 | 1434.22 ± 203.52 | 96.50 ± 13.62 |  | 6 | 19.70 ± 5.05 | 714.81 ± 80.74 |
| PL | 50 |  | 8 | 4.65 ± 1.37 |  | 7 | 421.42 ± 133.56 | 97.36 ± 17.90 |  | 4 | 15.35 ± 3.66 | 840.57 ± 321.53 |
| FCU | 50 |  | 8 | 19.25 ± 5.31 |  | 7 | 1133.52 ± 262.69 | 62.85 ± 11.47 |  | 5 | 17.83 ± 4.46 | 520.05 ± 104.52 |
| ECRL | 50 |  | 8 | 10.19 ± 1.49 |  | 8 | 1058.55 ± 200.75 | 104.35 ± 16.37 |  | 5 | 16.40 ± 1.50 | 835.68 ± 115.54 |
| ECRB | 50 |  | 8 | 16.81 ± 3.40 |  | 8 | 1396.22 ± 183.84 | 85.39 ± 16.70 |  | 7 | 18.33 ± 3.23 | 675.18 ± 74.57 |
| ECU | 50 |  | 8 | 14.16 ± 1.53 |  | 7 | 1169.10 ± 257.88 | 80.73 ± 13.24 |  | 7 | 18.13 ± 3.57 | 655.27 ± 103.66 |
| FDS-2 | 50 |  | 8 | 7.70 ± 1.18 |  | 7 | 726.11 ± 152.40 | 96.81 ± 14.27 |  | 5 | 17.01 ± 3.15 | 837.46 ± 188.82 |
| FDS-3 | 50 |  | 8 | 13.06 ± 1.52 |  | 8 | 1123.65 ± 198.73 | 85.96 ± 11.35 |  | 7 | 18.05 ± 3.43 | 677.26 ± 123.97 |
| FDS-4 | 50 |  | 8 | 10.41 ± 1.49 |  | 8 | 856.82 ± 176.56 | 82.49 ± 13.99 |  | 7 | 17.33 ± 2.12 | 662.91 ± 137.85 |
| FDS-5 | 50 |  | 8 | 2.72 ± 1.36 |  | 8 | 248.56 ± 121.73 | 92.36 ± 8.45 |  | 8 | 17.67 ± 3.62 | 724.39 ± 143.34 |
| FDP-2 | 50 |  | 8 | 9.33 ± 1.70 |  | 8 | 846.94 ± 215.90 | 90.59 ± 14.29 |  | 6 | 14.67 ± 4.34 | 772.28 ± 118.98 |
| FDP-3 | 50 |  | 8 | 12.47 ± 3.31 |  | 8 | 1096.82 ± 230.97 | 89.61 ± 15.38 |  | 5 | 15.68 ± 4.37 | 710.94 ± 225.21 |
| FDP-4 | 50 |  | 8 | 11.87 ± 2.25 |  | 8 | 964.08 ± 284.47 | 80.88 ± 13.87 |  | 8 | 17.27 ± 5.45 | 688.54 ± 144.99 |
| FDP-5 | 50 |  | 8 | 7.73 ± 1.22 |  | 8 | 615.40 ± 119.42 | 80.01 ± 10.95 |  | 7 | 16.40 ± 3.24 | 618.52 ± 99.68 |
| EIP | 50 |  | 8 | 4.17 ± 1.42 |  | 8 | 431.55 ± 88.45 | 109.47 ± 24.90 |  | 7 | 16.97 ± 2.64 | 883.68 ± 230.72 |
| EDC-2 | 50 |  | 8 | 3.64 ± 0.47 |  | 8 | 372.12 ± 110.30 | 102.62 ± 26.31 |  | 8 | 17.46 ± 4.64 | 793.44 ± 143.37 |
| EDC-3 | 50 |  | 8 | 6.72 ± 1.16 |  | 8 | 678.81 ± 132.55 | 104.08 ± 26.23 |  | 8 | 17.88 ± 4.16 | 783.97 ± 113.19 |
| EDC-4 | 50 |  | 8 | 6.53 ± 1.87 |  | 8 | 573.02 ± 127.59 | 91.23 ± 21.00 |  | 8 | 20.84 ± 3.38 | 632.83 ± 160.17 |
| EDC-5 | 50 |  | 6 | 2.23 ± 0.99 |  | 6 | 222.35 ± 80.12 | 107.75 ± 29.64 |  | 5 | 20.00 ± 2.73 | 639.12 ± 86.94 |
| EDM | 50 |  | 8 | 4.35 ± 1.61 |  | 8 | 432.61 ± 103.53 | 103.64 ± 17.22 |  | 8 | 20.79 ± 3.66 | 716.04 ± 150.15 |
| FPL | 50 |  | 8 | 11.47 ± 1.45 |  | 8 | 1011.29 ± 170.45 | 88.14 ± 8.85 |  | 3 | 18.89 ± 2.37 | 640.22 ± 142.62 |
| APL | 50 |  | 8 | 13.32 ± 2.54 |  | 7 | 1057.42 ± 173.76 | 77.00 ± 12.68 |  | 7 | 14.87 ± 2.38 | 722.93 ± 60.38 |
| EPB | 50 |  | 8 | 3.56 ± 2.44 |  | 8 | 406.43 ± 190.49 | 131.77 ± 38.97 |  | 7 | 15.58 ± 2.95 | 1020.05 ± 192.01 |
| EPL | 50 |  | 8 | 5.01 ± 1.25 |  | 8 | 440.70 ± 81.01 | 89.46 ± 10.79 |  | 8 | 16.35 ± 3.02 | 731.30 ± 142.89 |

Results – Pairwise Comparisons

Table A6. Statistical summary from mixed-effects linear regression analysis between human tendons and ligaments. Reported as the adjusted mean pairwise difference, or percentage change, between tendons relative to the reference tendon (right), with adjustment for regression model covariates. Significant differences after Šidák adjustment (adjusted P < 0.05) are annotated in bold with grey fill. *Key: ultrasound quantified cross-sectional area (CSA_US_); sulphated glycosaminoglycan (sGAG); hydroxyproline (Hyp); micrometer measured cross-sectional area (CSA_M_); ultimate tensile strength (UTS).*

| **Comparison** | **Length (mm)** | **Minor (mm)** | **Major (mm)** | **CSA_US_ (mm^2^)** | **sGAG (µg/mg)** | **Hyp (µg/mg)** | **CSA_M_ (mm^2^)** | **Failure Load (N)** | **UTS (MPa)** | **Failure Strain (%)** | **Modulus (MPa)** |
| --- | --- | --- | --- | --- | --- | --- | --- | --- | --- | --- | --- |
| IS vs SSP | 1 | -0.32 | 0.33 | -5% | **-2.7** | -7.0 | -1% | **139%** | 12.57 |  |  |
| SSC vs SSP | -2 | 0.34 | **6.89** | 32% | **-2.8** | -2.1 | **55%** | **183%** | 7.78 |  |  |
| LHBT vs SSP | **75** | **-3.52** | **-20.29** | **-88%** | **-3.9** | 12.4 | **-90%** | -14% | **73.36** |  |  |
| DBT vs SSP | **53** | **-3.21** | **-18.79** | **-85%** | **-6.0** | 9.6 | **-86%** | 35% | **75.00** |  |  |
| BrR vs SSP | **86** | **-4.65** | **-19.66** | **-91%** | **-6.5** | 8.1 | **-93%** | -31% | **77.66** |  |  |
| FCR vs SSP | **88** | **-3.60** | **-20.14** | **-89%** | **-6.6** | 15.0 | **-89%** | 25% | **88.01** |  |  |
| PL vs SSP | **121** | **-4.95** | **-21.85** | **-95%** | **-6.0** | 15.6 | **-97%** | **-63%** | **88.97** |  |  |
| FCU vs SSP | **120** | **-3.46** | **-18.18** | **-84%** | **-6.5** | 14.6 | **-86%** | 0% | **54.44** |  |  |
| ECRL vs SSP | **164** | **-4.42** | **-20.18** | **-91%** | **-6.3** | 17.1 | **-92%** | -8% | **95.86** |  |  |
| ECRB vs SSP | **100** | **-3.57** | **-19.72** | **-88%** | **-5.4** | 17.5 | **-88%** | 22% | **76.90** |  |  |
| ECU vs SSP | **61** | **-3.75** | **-19.76** | **-88%** | **-2.8** | 16.2 | **-89%** | -2% | **73.58** |  |  |
| FDS-2 vs SSP | **133** | **-4.36** | **-22.00** | **-94%** | **-5.8** | 18.3 | **-94%** | -35% | **88.85** |  |  |
| FDS-3 vs SSP | **148** | **-3.56** | **-21.50** | **-91%** | **-6.1** | **32.6** | **-90%** | -3% | **77.47** |  |  |
| FDS-4 vs SSP | **166** | **-3.78** | **-22.27** | **-93%** | **-5.4** | **25.4** | **-92%** | -26% | **74.00** |  |  |
| FDS-5 vs SSP | **118** | **-5.25** | **-23.54** | **-97%** | **-4.1** | 13.3 | **-98%** | **-79%** | **83.87** |  |  |
| FDP-2 vs SSP | **186** | **-4.00** | **-21.14** | **-92%** | **-5.0** | **20.4** | **-93%** | -28% | **82.10** |  |  |
| FDP-3 vs SSP | **154** | **-3.81** | **-21.10** | **-91%** | **-4.7** | **37.8** | **-91%** | -6% | **81.12** |  |  |
| FDP-4 vs SSP | **124** | **-4.00** | **-20.75** | **-91%** | **-4.4** | **24.0** | **-91%** | -17% | **72.39** |  |  |
| FDP-5 vs SSP | **162** | **-4.23** | **-22.19** | **-94%** | **-3.2** | 15.3 | **-94%** | **-47%** | **71.52** |  |  |
| EIP vs SSP | **94** | **-5.08** | **-21.85** | **-95%** | **-5.5** | 0.3 | **-97%** | **-63%** | **100.98** |  |  |
| EDC-2 vs SSP | **151** | **-5.12** | **-22.64** | **-96%** | **-4.1** | 3.1 | **-97%** | **-68%** | **94.13** |  |  |
| EDC-3 vs SSP | **203** | **-4.44** | **-22.21** | **-94%** | **-5.3** | -2.4 | **-95%** | **-41%** | **95.59** |  |  |
| EDC-4 vs SSP | **155** | **-4.47** | **-22.21** | **-94%** | **-3.7** | 1.8 | **-95%** | **-50%** | **82.74** |  |  |
| EDC-5 vs SSP | **109** | **-5.26** | **-23.39** | **-97%** | **-3.4** | -1.4 | **-98%** | **-81%** | **97.88** |  |  |
| EDM vs SSP | **107** | **-4.83** | **-22.82** | **-96%** | **-3.9** | 8.0 | **-97%** | **-63%** | **95.15** |  |  |
| FPL vs SSP | **125** | **-3.38** | **-21.90** | **-91%** | **-5.4** | 17.8 | **-91%** | -12% | **79.65** |  |  |
| APL vs SSP | **68** | **-3.86** | **-19.83** | **-89%** | **-5.1** | 15.1 | **-90%** | -10% | **69.84** |  |  |
| EPB vs SSP | **78** | **-4.81** | **-22.81** | **-96%** | **-3.2** | 12.9 | **-98%** | **-66%** | **123.28** |  |  |
| EPL vs SSP | **119** | **-4.91** | **-21.73** | **-95%** | **-4.1** | 15.4 | **-96%** | **-62%** | **80.97** |  |  |
| SSC vs IS | -3 | 0.67 | **6.56** | 38% | 0.0 | 4.9 | **57%** | 18% | -4.79 |  |  |
| LHBT vs IS | **74** | **-3.19** | **-20.62** | **-88%** | -1.2 | **19.4** | **-90%** | **-64%** | **60.79** |  |  |
| DBT vs IS | **52** | **-2.88** | **-19.12** | **-84%** | **-3.3** | 16.6 | **-86%** | **-44%** | **62.43** |  |  |
| BrR vs IS | **85** | **-4.32** | **-19.99** | **-91%** | **-3.7** | 15.1 | **-93%** | **-71%** | **65.09** |  |  |
| FCR vs IS | **88** | **-3.28** | **-20.46** | **-88%** | **-3.9** | **22.0** | **-89%** | **-48%** | **75.44** |  |  |
| PL vs IS | **121** | **-4.63** | **-22.18** | **-95%** | **-3.3** | **22.6** | **-96%** | **-85%** | **76.40** |  |  |
| FCU vs IS | **119** | **-3.13** | **-18.51** | **-84%** | **-3.8** | **21.6** | **-86%** | **-58%** | **41.87** |  |  |
| ECRL vs IS | **163** | **-4.09** | **-20.50** | **-91%** | **-3.6** | **24.1** | **-92%** | **-62%** | **83.29** |  |  |
| ECRB vs IS | **99** | **-3.25** | **-20.05** | **-87%** | **-2.6** | **24.5** | **-87%** | **-49%** | **64.33** |  |  |
| ECU vs IS | **61** | **-3.42** | **-20.08** | **-88%** | -0.1 | **23.2** | **-89%** | **-59%** | **61.01** |  |  |
| FDS-2 vs IS | **132** | **-4.04** | **-22.33** | **-93%** | **-3.1** | **25.3** | **-94%** | **-73%** | **76.28** |  |  |
| FDS-3 vs IS | **147** | **-3.23** | **-21.83** | **-90%** | **-3.4** | **39.6** | **-90%** | **-59%** | **64.90** |  |  |
| FDS-4 vs IS | **165** | **-3.45** | **-22.60** | **-92%** | **-2.7** | **32.4** | **-92%** | **-69%** | **61.43** |  |  |
| FDS-5 vs IS | **118** | **-4.92** | **-23.87** | **-97%** | -1.4 | **20.3** | **-98%** | **-91%** | **71.30** |  |  |
| FDP-2 vs IS | **185** | **-3.67** | **-21.47** | **-91%** | **-2.3** | **27.5** | **-93%** | **-70%** | **69.53** |  |  |
| FDP-3 vs IS | **153** | **-3.49** | **-21.43** | **-90%** | -1.9 | **44.8** | **-91%** | **-61%** | **68.55** |  |  |
| FDP-4 vs IS | **124** | **-3.67** | **-21.07** | **-90%** | -1.6 | **31.0** | **-91%** | **-66%** | **59.82** |  |  |
| FDP-5 vs IS | **161** | **-3.90** | **-22.52** | **-93%** | -0.4 | **22.3** | **-94%** | **-78%** | **58.95** |  |  |
| EIP vs IS | **94** | **-4.75** | **-22.18** | **-95%** | **-2.8** | 7.4 | **-97%** | **-85%** | **88.41** |  |  |
| EDC-2 vs IS | **151** | **-4.80** | **-22.97** | **-96%** | -1.4 | 10.1 | **-97%** | **-87%** | **81.56** |  |  |
| EDC-3 vs IS | **202** | **-4.12** | **-22.54** | **-94%** | **-2.5** | 4.6 | **-95%** | **-75%** | **83.02** |  |  |
| EDC-4 vs IS | **154** | **-4.15** | **-22.54** | **-94%** | -0.9 | 8.8 | **-95%** | **-79%** | **70.17** |  |  |
| EDC-5 vs IS | **108** | **-4.94** | **-23.72** | **-97%** | -0.7 | 5.6 | **-98%** | **-92%** | **85.31** |  |  |
| EDM vs IS | **106** | **-4.51** | **-23.15** | **-96%** | -1.1 | 15.0 | **-97%** | **-85%** | **82.58** |  |  |
| FPL vs IS | **124** | **-3.06** | **-22.23** | **-91%** | **-2.7** | **24.8** | **-91%** | **-63%** | **67.08** |  |  |
| APL vs IS | **67** | **-3.54** | **-20.16** | **-88%** | **-2.4** | **22.1** | **-90%** | **-63%** | **57.27** |  |  |
| EPB vs IS | **77** | **-4.48** | **-23.13** | **-95%** | -0.4 | **19.9** | **-97%** | **-86%** | **110.71** |  |  |
| EPL vs IS | **119** | **-4.59** | **-22.06** | **-95%** | -1.4 | **22.4** | **-96%** | **-84%** | **68.40** |  |  |
| LHBT vs SSC | **77** | **-3.86** | **-27.18** | **-91%** | -1.2 | 14.5 | **-94%** | **-70%** | **65.59** |  |  |
| DBT vs SSC | **54** | **-3.55** | **-25.68** | **-88%** | **-3.3** | 11.7 | **-91%** | **-52%** | **67.22** |  |  |
| BrR vs SSC | **88** | **-4.99** | **-26.55** | **-93%** | **-3.7** | 10.3 | **-95%** | **-76%** | **69.89** |  |  |
| FCR vs SSC | **90** | **-3.95** | **-27.03** | **-91%** | **-3.9** | 17.1 | **-93%** | **-56%** | **80.24** |  |  |
| PL vs SSC | **123** | **-5.30** | **-28.74** | **-96%** | **-3.3** | 17.7 | **-98%** | **-87%** | **81.19** |  |  |
| FCU vs SSC | **122** | **-3.80** | **-25.07** | **-88%** | **-3.8** | 16.7 | **-91%** | **-65%** | **46.66** |  |  |
| ECRL vs SSC | **166** | **-4.76** | **-27.07** | **-93%** | **-3.5** | **19.2** | **-95%** | **-68%** | **88.09** |  |  |
| ECRB vs SSC | **102** | **-3.92** | **-26.61** | **-91%** | **-2.6** | **19.6** | **-92%** | **-57%** | **69.12** |  |  |
| ECU vs SSC | **63** | **-4.09** | **-26.65** | **-91%** | 0.0 | 18.3 | **-93%** | **-65%** | **65.80** |  |  |
| FDS-2 vs SSC | **134** | **-4.71** | **-28.89** | **-95%** | **-3.0** | **20.5** | **-96%** | **-77%** | **81.08** |  |  |
| FDS-3 vs SSC | **149** | **-3.90** | **-28.39** | **-93%** | **-3.4** | **34.7** | **-94%** | **-66%** | **69.69** |  |  |
| FDS-4 vs SSC | **168** | **-4.12** | **-29.16** | **-95%** | **-2.7** | **27.5** | **-95%** | **-74%** | **66.22** |  |  |
| FDS-5 vs SSC | **120** | **-5.59** | **-30.43** | **-98%** | -1.3 | 15.4 | **-99%** | **-93%** | **76.09** |  |  |
| FDP-2 vs SSC | **188** | **-4.34** | **-28.03** | **-94%** | **-2.3** | **22.6** | **-96%** | **-74%** | **74.32** |  |  |
| FDP-3 vs SSC | **156** | **-4.16** | **-27.99** | **-93%** | -1.9 | **40.0** | **-94%** | **-67%** | **73.34** |  |  |
| FDP-4 vs SSC | **126** | **-4.34** | **-27.64** | **-93%** | -1.6 | **26.1** | **-94%** | **-71%** | **64.62** |  |  |
| FDP-5 vs SSC | **164** | **-4.57** | **-29.08** | **-95%** | -0.4 | 17.4 | **-96%** | **-81%** | **63.74** |  |  |
| EIP vs SSC | **96** | **-5.42** | **-28.74** | **-96%** | **-2.7** | 2.5 | **-98%** | **-87%** | **93.21** |  |  |
| EDC-2 vs SSC | **153** | **-5.47** | **-29.53** | **-97%** | -1.4 | 5.3 | **-98%** | **-89%** | **86.36** |  |  |
| EDC-3 vs SSC | **204** | **-4.79** | **-29.10** | **-96%** | **-2.5** | -0.3 | **-97%** | **-79%** | **87.82** |  |  |
| EDC-4 vs SSC | **157** | **-4.82** | **-29.10** | **-96%** | -0.9 | 3.9 | **-97%** | **-82%** | **74.97** |  |  |
| EDC-5 vs SSC | **111** | **-5.60** | **-30.28** | **-98%** | -0.6 | 0.7 | **-99%** | **-93%** | **90.11** |  |  |
| EDM vs SSC | **109** | **-5.18** | **-29.71** | **-97%** | -1.1 | 10.1 | **-98%** | **-87%** | **87.38** |  |  |
| FPL vs SSC | **127** | **-3.73** | **-28.79** | **-93%** | **-2.7** | **19.9** | **-94%** | **-69%** | **71.87** |  |  |
| APL vs SSC | **69** | **-4.21** | **-26.72** | **-92%** | **-2.4** | 17.2 | **-94%** | **-68%** | **62.06** |  |  |
| EPB vs SSC | **79** | **-5.15** | **-29.70** | **-97%** | -0.4 | 15.0 | **-98%** | **-88%** | **115.51** |  |  |
| EPL vs SSC | **121** | **-5.26** | **-28.62** | **-96%** | -1.4 | 17.5 | **-98%** | **-87%** | **73.19** |  |  |
| DBT vs LHBT | -23 | 0.31 | 1.49 | 32% | -2.1 | -2.8 | 41% | **58%** | 1.64 | 0.04 | -84.64 |
| BrR vs LHBT | 11 | **-1.13** | 0.63 | -26% | **-2.5** | -4.3 | -29% | -19% | 4.30 | 0.00 | 91.14 |
| FCR vs LHBT | 13 | -0.08 | 0.15 | -1% | **-2.7** | 2.6 | 12% | 46% | 14.65 | 0.02 | 88.63 |
| PL vs LHBT | **46** | **-1.43** | -1.56 | **-56%** | -2.1 | 3.2 | **-65%** | **-57%** | 15.61 | -0.01 | 202.72 |
| FCU vs LHBT | **45** | 0.06 | 2.11 | 34% | **-2.6** | 2.2 | 42% | 17% | -18.93 | 0.01 | -108.82 |
| ECRL vs LHBT | **89** | -0.90 | 0.11 | -26% | -2.4 | 4.7 | -24% | 7% | 22.50 | 0.00 | 208.45 |
| ECRB vs LHBT | 25 | -0.05 | 0.57 | 6% | -1.4 | 5.1 | 24% | 42% | 3.53 | 0.01 | 53.81 |
| ECU vs LHBT | -14 | -0.23 | 0.53 | 1% | 1.1 | 3.8 | 6% | 15% | 0.21 | 0.01 | 39.70 |
| FDS-2 vs LHBT | **58** | -0.85 | -1.71 | **-46%** | -1.9 | 5.9 | **-42%** | -24% | 15.49 | 0.00 | 203.03 |
| FDS-3 vs LHBT | **73** | -0.04 | -1.22 | -20% | -2.2 | **20.2** | -2% | 14% | 4.11 | 0.01 | 53.81 |
| FDS-4 vs LHBT | **91** | -0.26 | -1.99 | **-38%** | -1.5 | 13.0 | -21% | -14% | 0.63 | 0.00 | 39.46 |
| FDS-5 vs LHBT | **43** | **-1.73** | **-3.25** | **-76%** | -0.2 | 0.9 | **-80%** | **-76%** | 10.50 | 0.00 | 104.74 |
| FDP-2 vs LHBT | **111** | -0.48 | -0.85 | -27% | -1.1 | 8.0 | -31% | -15% | 8.74 | -0.03 | 140.12 |
| FDP-3 vs LHBT | **79** | -0.29 | -0.82 | -21% | -0.7 | **25.4** | -7% | 10% | 7.76 | -0.02 | 76.43 |
| FDP-4 vs LHBT | **49** | -0.48 | -0.46 | -20% | -0.4 | 11.6 | -12% | -3% | -0.97 | 0.00 | 68.90 |
| FDP-5 vs LHBT | **87** | -0.71 | -1.90 | **-46%** | 0.8 | 2.9 | **-42%** | **-38%** | -1.85 | -0.01 | 2.95 |
| EIP vs LHBT | 19 | **-1.56** | -1.57 | **-58%** | -1.6 | -12.1 | **-69%** | **-57%** | 27.62 | 0.00 | **262.31** |
| EDC-2 vs LHBT | **76** | **-1.61** | -2.35 | **-67%** | -0.2 | -9.3 | **-73%** | **-62%** | 20.77 | 0.00 | 173.79 |
| EDC-3 vs LHBT | **128** | -0.92 | -1.92 | **-50%** | -1.3 | -14.8 | **-50%** | -31% | 22.23 | 0.00 | 164.33 |
| EDC-4 vs LHBT | **80** | -0.95 | -1.92 | **-50%** | 0.3 | -10.6 | **-52%** | **-42%** | 9.38 | 0.03 | 13.18 |
| EDC-5 vs LHBT | 34 | **-1.74** | **-3.10** | **-76%** | 0.5 | -13.8 | **-83%** | **-78%** | 24.52 | 0.02 | 36.55 |
| EDM vs LHBT | 32 | **-1.31** | -2.53 | **-65%** | 0.1 | -4.4 | **-68%** | **-57%** | 21.79 | 0.03 | 96.39 |
| FPL vs LHBT | **50** | 0.14 | -1.61 | -24% | -1.5 | 5.4 | -14% | 2% | 6.28 | 0.02 | 23.92 |
| APL vs LHBT | -8 | -0.35 | 0.46 | -4% | -1.2 | 2.7 | 0% | 5% | -3.53 | -0.03 | 107.36 |
| EPB vs LHBT | 3 | **-1.29** | **-2.52** | **-62%** | 0.8 | 0.5 | **-75%** | **-60%** | **49.92** | -0.02 | **391.47** |
| EPL vs LHBT | **44** | **-1.40** | -1.44 | **-55%** | -0.2 | 3.0 | **-63%** | **-55%** | 7.60 | -0.01 | 111.66 |
| BrR vs DBT | 33 | **-1.44** | -0.87 | **-44%** | -0.4 | -1.4 | **-50%** | **-49%** | 2.66 | -0.05 | 175.78 |
| FCR vs DBT | 36 | -0.40 | -1.34 | -25% | -0.6 | 5.4 | -21% | -7% | 13.01 | -0.02 | 173.27 |
| PL vs DBT | **69** | **-1.75** | **-3.06** | **-67%** | 0.0 | 6.0 | **-75%** | **-73%** | 13.97 | -0.06 | 287.36 |
| FCU vs DBT | **68** | -0.25 | 0.61 | 2% | -0.5 | 5.0 | 0% | -26% | -20.56 | -0.03 | -24.19 |
| ECRL vs DBT | **111** | **-1.21** | -1.38 | **-44%** | -0.3 | 7.5 | **-46%** | -32% | 20.86 | -0.05 | **293.08** |
| ECRB vs DBT | **48** | -0.37 | -0.93 | -20% | 0.7 | 7.9 | -12% | -10% | 1.90 | -0.03 | 138.44 |
| ECU vs DBT | 9 | -0.54 | -0.96 | -24% | **3.2** | 6.6 | -25% | -27% | -1.42 | -0.04 | 124.34 |
| FDS-2 vs DBT | **80** | **-1.16** | **-3.21** | **-59%** | 0.2 | 8.8 | **-59%** | **-52%** | 13.85 | -0.04 | **287.67** |
| FDS-3 vs DBT | **95** | -0.35 | **-2.71** | **-39%** | -0.1 | **23.0** | -30% | -28% | 2.47 | -0.04 | 138.45 |
| FDS-4 vs DBT | **113** | -0.57 | **-3.48** | **-53%** | 0.6 | 15.8 | **-44%** | **-45%** | -1.00 | -0.05 | 124.10 |
| FDS-5 vs DBT | **66** | **-2.04** | **-4.75** | **-82%** | 1.9 | 3.8 | **-86%** | **-84%** | 8.87 | -0.04 | 189.38 |
| FDP-2 vs DBT | **133** | -0.79 | -2.34 | **-44%** | 1.0 | 10.9 | **-51%** | **-46%** | 7.10 | **-0.07** | 224.76 |
| FDP-3 vs DBT | **101** | -0.61 | -2.31 | **-40%** | 1.4 | **28.3** | -34% | -30% | 6.12 | -0.06 | 161.06 |
| FDP-4 vs DBT | **72** | -0.79 | -1.95 | **-40%** | 1.7 | 14.4 | **-38%** | **-39%** | -2.61 | -0.05 | 153.53 |
| FDP-5 vs DBT | **109** | **-1.02** | **-3.40** | **-59%** | **2.9** | 5.7 | **-59%** | **-61%** | -3.48 | **-0.06** | 87.59 |
| EIP vs DBT | **42** | **-1.87** | **-3.06** | **-69%** | 0.5 | -9.2 | **-78%** | **-72%** | 25.98 | -0.05 | **346.95** |
| EDC-2 vs DBT | **99** | **-1.92** | **-3.85** | **-75%** | 1.9 | -6.4 | **-81%** | **-76%** | 19.13 | -0.05 | **258.43** |
| EDC-3 vs DBT | **150** | **-1.24** | **-3.42** | **-62%** | 0.8 | -12.0 | **-64%** | **-56%** | 20.59 | -0.04 | **248.97** |
| EDC-4 vs DBT | **103** | **-1.27** | **-3.42** | **-62%** | **2.4** | -7.8 | **-66%** | **-63%** | 7.74 | -0.01 | 97.82 |
| EDC-5 vs DBT | **56** | **-2.05** | **-4.60** | **-82%** | **2.6** | -11.0 | **-88%** | **-86%** | 22.88 | -0.03 | 121.19 |
| EDM vs DBT | **54** | **-1.63** | **-4.02** | **-74%** | 2.2 | -1.6 | **-77%** | **-72%** | 20.15 | -0.01 | 181.03 |
| FPL vs DBT | **73** | -0.18 | **-3.11** | **-43%** | 0.6 | 8.2 | **-39%** | **-35%** | 4.65 | -0.02 | 108.56 |
| APL vs DBT | 15 | -0.66 | -1.04 | -27% | 0.9 | 5.5 | -29% | -33% | -5.16 | **-0.07** | 192.00 |
| EPB vs DBT | 25 | **-1.60** | **-4.01** | **-71%** | **2.9** | 3.4 | **-82%** | **-75%** | **48.28** | **-0.06** | **476.10** |
| EPL vs DBT | **67** | **-1.71** | **-2.94** | **-66%** | 1.9 | 5.8 | **-74%** | **-72%** | 5.97 | **-0.06** | 196.30 |
| FCR vs BrR | 3 | **1.05** | -0.48 | 33% | -0.2 | 6.9 | **58%** | **80%** | 10.35 | 0.03 | -2.51 |
| PL vs BrR | 36 | -0.30 | -2.19 | **-41%** | 0.5 | 7.4 | **-50%** | **-47%** | 11.31 | -0.01 | 111.58 |
| FCU vs BrR | 34 | **1.19** | 1.48 | **81%** | 0.0 | 6.4 | **100%** | 44% | -23.23 | 0.01 | -199.97 |
| ECRL vs BrR | **78** | 0.23 | -0.51 | 0% | 0.2 | 9.0 | 8% | 33% | 18.20 | 0.00 | 117.30 |
| ECRB vs BrR | 14 | **1.08** | -0.06 | 43% | 1.1 | 9.3 | **76%** | **76%** | -0.77 | 0.02 | -37.34 |
| ECU vs BrR | -24 | 0.90 | -0.10 | 36% | **3.7** | 8.0 | 50% | 42% | -4.09 | 0.01 | -51.44 |
| FDS-2 vs BrR | **47** | 0.28 | -2.34 | -27% | 0.7 | 10.2 | -18% | -6% | 11.19 | 0.01 | 111.89 |
| FDS-3 vs BrR | **62** | **1.09** | -1.84 | 8% | 0.4 | **24.4** | 39% | 41% | -0.19 | 0.01 | -37.33 |
| FDS-4 vs BrR | **80** | 0.87 | **-2.61** | -16% | 1.0 | 17.2 | 11% | 7% | -3.67 | 0.00 | -51.68 |
| FDS-5 vs BrR | 33 | -0.60 | **-3.88** | **-68%** | **2.4** | 5.2 | **-72%** | **-70%** | 6.20 | 0.01 | 13.60 |
| FDP-2 vs BrR | **100** | 0.65 | -1.48 | -1% | 1.4 | 12.3 | -2% | 5% | 4.44 | -0.02 | 48.97 |
| FDP-3 vs BrR | **68** | 0.84 | -1.44 | 7% | 1.8 | **29.7** | 31% | 37% | 3.46 | -0.01 | -14.72 |
| FDP-4 vs BrR | **39** | 0.65 | -1.08 | 8% | 2.1 | 15.9 | 25% | 20% | -5.27 | 0.00 | -22.25 |
| FDP-5 vs BrR | **76** | 0.42 | **-2.53** | -27% | **3.3** | 7.2 | -18% | -23% | -6.15 | -0.01 | -88.20 |
| EIP vs BrR | 9 | -0.43 | -2.19 | **-44%** | 1.0 | -7.8 | **-56%** | **-46%** | 23.32 | 0.00 | 171.17 |
| EDC-2 vs BrR | **66** | -0.48 | **-2.98** | **-56%** | **2.4** | -5.0 | **-61%** | **-53%** | 16.47 | 0.00 | 82.65 |
| EDC-3 vs BrR | **117** | 0.21 | **-2.55** | -32% | 1.2 | -10.5 | -29% | -15% | 17.93 | 0.01 | 73.19 |
| EDC-4 vs BrR | **69** | 0.18 | -2.55 | -32% | **2.8** | -6.3 | -32% | -28% | 5.08 | 0.04 | -77.96 |
| EDC-5 vs BrR | 23 | -0.61 | **-3.73** | **-68%** | **3.1** | -9.5 | **-76%** | **-72%** | 20.22 | 0.02 | -54.59 |
| EDM vs BrR | 21 | -0.18 | **-3.16** | **-53%** | **2.6** | -0.1 | **-55%** | **-46%** | 17.49 | 0.04 | 5.25 |
| FPL vs BrR | **39** | **1.27** | -2.24 | 2% | 1.0 | 9.6 | 21% | 27% | 1.99 | 0.03 | -67.22 |
| APL vs BrR | -18 | 0.78 | -0.17 | 30% | 1.3 | 6.9 | 42% | 30% | -7.82 | -0.02 | 16.22 |
| EPB vs BrR | -8 | -0.16 | **-3.14** | **-48%** | **3.3** | 4.8 | **-65%** | **-51%** | **45.62** | -0.01 | **300.32** |
| EPL vs BrR | 34 | -0.27 | -2.07 | **-39%** | 2.3 | 7.3 | **-48%** | **-45%** | 3.30 | -0.01 | 20.52 |
| PL vs FCR | 33 | **-1.35** | -1.71 | **-56%** | 0.6 | 0.6 | **-69%** | **-70%** | 0.96 | -0.03 | 114.08 |
| FCU vs FCR | 32 | 0.14 | 1.96 | 36% | 0.1 | -0.5 | 26% | -20% | **-33.58** | -0.01 | -197.46 |
| ECRL vs FCR | **76** | -0.82 | -0.04 | -25% | 0.3 | 2.1 | -32% | -27% | 7.85 | -0.02 | 119.81 |
| ECRB vs FCR | 12 | 0.03 | 0.42 | 8% | 1.3 | 2.5 | 11% | -3% | -11.12 | -0.01 | -34.83 |
| ECU vs FCR | -27 | -0.15 | 0.38 | 2% | **3.8** | 1.2 | -5% | -21% | -14.44 | -0.02 | -48.93 |
| FDS-2 vs FCR | **44** | -0.76 | -1.86 | **-45%** | 0.8 | 3.3 | **-48%** | **-48%** | 0.84 | -0.02 | 114.40 |
| FDS-3 vs FCR | **59** | 0.04 | -1.37 | -19% | 0.5 | 17.6 | -12% | -22% | -10.54 | -0.02 | -34.83 |
| FDS-4 vs FCR | **78** | -0.18 | -2.14 | **-37%** | 1.2 | 10.4 | -30% | **-41%** | -14.02 | -0.02 | -49.18 |
| FDS-5 vs FCR | 30 | **-1.64** | **-3.40** | **-76%** | **2.5** | -1.7 | **-82%** | **-83%** | -4.15 | -0.02 | 16.11 |
| FDP-2 vs FCR | **98** | -0.39 | -1.00 | -26% | 1.6 | 5.4 | **-38%** | **-42%** | -5.91 | -0.05 | 51.48 |
| FDP-3 vs FCR | **66** | -0.21 | -0.97 | -20% | 2.0 | **22.8** | -17% | -24% | -6.89 | -0.04 | -12.21 |
| FDP-4 vs FCR | 36 | -0.40 | -0.61 | -19% | 2.3 | 9.0 | -21% | **-34%** | -15.62 | -0.02 | -19.74 |
| FDP-5 vs FCR | **74** | -0.63 | -2.06 | **-45%** | **3.5** | 0.3 | **-49%** | **-57%** | -16.49 | -0.03 | -85.69 |
| EIP vs FCR | 6 | **-1.48** | -1.72 | **-58%** | 1.1 | -14.7 | **-72%** | **-70%** | 12.97 | -0.02 | 173.68 |
| EDC-2 vs FCR | **63** | **-1.52** | **-2.50** | **-67%** | **2.5** | -11.9 | **-76%** | **-74%** | 6.12 | -0.02 | 85.16 |
| EDC-3 vs FCR | **114** | -0.84 | -2.07 | **-49%** | 1.4 | -17.4 | **-55%** | **-53%** | 7.58 | -0.02 | 75.70 |
| EDC-4 vs FCR | **67** | -0.87 | -2.07 | **-49%** | **3.0** | -13.2 | **-57%** | **-60%** | -5.27 | 0.01 | -75.45 |
| EDC-5 vs FCR | 21 | **-1.66** | **-3.26** | **-76%** | **3.2** | -16.4 | **-85%** | **-85%** | 9.87 | 0.00 | -52.08 |
| EDM vs FCR | 19 | **-1.23** | **-2.68** | **-65%** | **2.8** | -7.0 | **-71%** | **-70%** | 7.14 | 0.01 | 7.76 |
| FPL vs FCR | **37** | 0.22 | -1.77 | -23% | 1.2 | 2.7 | -23% | -30% | -8.36 | 0.00 | -64.72 |
| APL vs FCR | -21 | -0.26 | 0.31 | -3% | 1.5 | 0.1 | -11% | -28% | -18.17 | -0.05 | 18.73 |
| EPB vs FCR | -11 | **-1.21** | **-2.67** | **-61%** | **3.5** | -2.1 | **-78%** | **-73%** | **35.27** | -0.04 | **302.83** |
| EPL vs FCR | 31 | **-1.31** | -1.60 | **-54%** | **2.5** | 0.4 | **-67%** | **-69%** | -7.05 | -0.03 | 23.03 |
| FCU vs PL | -1 | **1.50** | **3.67** | **205%** | -0.5 | -1.0 | **304%** | **171%** | **-34.53** | 0.02 | -311.54 |
| ECRL vs PL | **43** | 0.54 | 1.67 | **69%** | -0.3 | 1.5 | **117%** | **148%** | 6.89 | 0.01 | 5.73 |
| ECRB vs PL | -21 | **1.38** | 2.13 | **142%** | 0.7 | 1.9 | **255%** | **230%** | -12.07 | 0.02 | -148.91 |
| ECU vs PL | **-60** | **1.20** | 2.09 | **130%** | **3.2** | 0.6 | **202%** | **167%** | -15.39 | 0.02 | -163.02 |
| FDS-2 vs PL | 11 | 0.59 | -0.15 | 23% | 0.2 | 2.8 | **65%** | **77%** | -0.12 | 0.02 | 0.31 |
| FDS-3 vs PL | 26 | **1.40** | 0.35 | **82%** | -0.1 | 17.0 | **181%** | **164%** | -11.50 | 0.02 | -148.91 |
| FDS-4 vs PL | **44** | **1.17** | -0.43 | 42% | 0.6 | 9.8 | **125%** | **100%** | -14.97 | 0.01 | -163.26 |
| FDS-5 vs PL | -3 | -0.29 | -1.69 | **-45%** | 1.9 | -2.2 | **-43%** | **-43%** | -5.10 | 0.01 | -97.97 |
| FDP-2 vs PL | **64** | **0.96** | 0.71 | **67%** | 1.0 | 4.9 | **98%** | **96%** | -6.87 | -0.02 | -62.60 |
| FDP-3 vs PL | 33 | **1.14** | 0.75 | **80%** | 1.3 | **22.3** | **165%** | **156%** | -7.85 | 0.00 | -126.29 |
| FDP-4 vs PL | 3 | **0.95** | 1.10 | **82%** | 1.7 | 8.4 | **152%** | **124%** | -16.58 | 0.01 | -133.82 |
| FDP-5 vs PL | **41** | 0.73 | -0.34 | 23% | **2.9** | -0.3 | **64%** | 44% | -17.45 | 0.00 | -199.77 |
| EIP vs PL | -27 | -0.12 | -0.01 | -5% | 0.5 | -15.2 | -10% | 1% | 12.01 | 0.01 | 59.59 |
| EDC-2 vs PL | 30 | -0.17 | -0.79 | -25% | 1.9 | -12.4 | -22% | -12% | 5.16 | 0.01 | -28.93 |
| EDC-3 vs PL | **81** | 0.51 | -0.36 | 14% | 0.8 | -18.0 | 44% | **60%** | 6.62 | 0.01 | -38.39 |
| EDC-4 vs PL | 34 | 0.48 | -0.36 | 14% | **2.4** | -13.8 | 37% | 36% | -6.23 | 0.04 | -189.54 |
| EDC-5 vs PL | -12 | -0.31 | -1.54 | **-46%** | **2.6** | -17.0 | **-52%** | **-48%** | 8.91 | 0.03 | -166.17 |
| EDM vs PL | -14 | 0.12 | -0.97 | -21% | 2.2 | -7.6 | -9% | 1% | 6.18 | 0.04 | -106.33 |
| FPL vs PL | 4 | **1.57** | -0.05 | **72%** | 0.6 | 2.2 | **145%** | **138%** | -9.32 | 0.03 | -178.80 |
| APL vs PL | **-54** | **1.09** | 2.02 | **119%** | 0.9 | -0.5 | **186%** | **143%** | -19.13 | -0.02 | -95.36 |
| EPB vs PL | **-44** | 0.15 | -0.96 | -13% | **2.9** | -2.6 | -29% | -7% | **34.31** | -0.01 | 188.75 |
| EPL vs PL | -2 | 0.04 | 0.12 | 3% | 1.9 | -0.2 | 5% | 3% | -8.00 | 0.00 | -91.06 |
| ECRL vs FCU | **44** | **-0.96** | -2.00 | **-45%** | 0.2 | 2.6 | **-46%** | -8% | **41.43** | -0.01 | **317.27** |
| ECRB vs FCU | -20 | -0.12 | -1.54 | -21% | 1.2 | 2.9 | -12% | 22% | 22.46 | 0.00 | 162.63 |
| ECU vs FCU | **-59** | -0.29 | -1.58 | -25% | **3.7** | 1.6 | -25% | -1% | 19.14 | 0.00 | 148.53 |
| FDS-2 vs FCU | 13 | -0.91 | **-3.82** | **-60%** | 0.7 | 3.8 | **-59%** | -35% | **34.42** | -0.01 | **311.86** |
| FDS-3 vs FCU | 28 | -0.10 | **-3.32** | **-40%** | 0.4 | 18.0 | -30% | -3% | 23.03 | 0.00 | 162.63 |
| FDS-4 vs FCU | **46** | -0.32 | **-4.09** | **-54%** | 1.1 | 10.8 | **-44%** | -26% | 19.56 | -0.01 | 148.28 |
| FDS-5 vs FCU | -2 | **-1.79** | **-5.36** | **-82%** | **2.4** | -1.2 | **-86%** | **-79%** | 29.43 | -0.01 | 213.57 |
| FDP-2 vs FCU | **66** | -0.54 | **-2.96** | **-45%** | 1.5 | 5.9 | **-51%** | -27% | 27.66 | -0.04 | 248.94 |
| FDP-3 vs FCU | 34 | -0.36 | **-2.92** | **-41%** | 1.9 | **23.3** | -34% | -5% | 26.68 | -0.03 | 185.25 |
| FDP-4 vs FCU | 4 | -0.54 | **-2.57** | **-41%** | 2.2 | 9.5 | **-38%** | -17% | 17.96 | -0.01 | 177.72 |
| FDP-5 vs FCU | **42** | -0.77 | **-4.01** | **-60%** | **3.4** | 0.8 | **-59%** | **-47%** | 17.08 | -0.02 | 111.77 |
| EIP vs FCU | -26 | **-1.62** | **-3.67** | **-69%** | 1.0 | -14.2 | **-78%** | **-63%** | **46.55** | -0.01 | **371.14** |
| EDC-2 vs FCU | 31 | **-1.67** | **-4.46** | **-76%** | **2.4** | -11.4 | **-81%** | **-68%** | **39.70** | -0.01 | **282.62** |
| EDC-3 vs FCU | **83** | **-0.98** | **-4.03** | **-63%** | 1.3 | -17.0 | **-65%** | **-41%** | **41.16** | -0.01 | **273.16** |
| EDC-4 vs FCU | 35 | **-1.02** | **-4.03** | **-63%** | **2.9** | -12.8 | **-66%** | **-50%** | 28.31 | 0.02 | 122.01 |
| EDC-5 vs FCU | -11 | **-1.80** | **-5.21** | **-82%** | **3.1** | -15.9 | **-88%** | **-81%** | **43.45** | 0.01 | 145.38 |
| EDM vs FCU | -13 | **-1.38** | **-4.64** | **-74%** | **2.7** | -6.5 | **-77%** | **-63%** | **40.72** | 0.02 | 205.22 |
| FPL vs FCU | 5 | 0.07 | **-3.72** | **-44%** | 1.1 | 3.2 | **-39%** | -12% | 25.21 | 0.01 | 132.74 |
| APL vs FCU | **-53** | -0.41 | -1.65 | -28% | 1.4 | 0.5 | -29% | -10% | 15.40 | -0.04 | 216.19 |
| EPB vs FCU | **-43** | **-1.35** | **-4.63** | **-72%** | **3.4** | -1.6 | **-82%** | **-66%** | **68.85** | -0.03 | **500.29** |
| EPL vs FCU | -1 | **-1.46** | **-3.55** | **-66%** | **2.4** | 0.8 | **-74%** | **-62%** | 26.53 | -0.02 | 220.49 |
| ECRB vs ECRL | **-64** | 0.84 | 0.45 | 43% | 0.9 | 0.4 | **64%** | 33% | -18.97 | 0.01 | -154.64 |
| ECU vs ECRL | **-103** | 0.67 | 0.42 | 36% | **3.5** | -0.9 | 39% | 7% | -22.29 | 0.01 | -168.74 |
| FDS-2 vs ECRL | -31 | 0.05 | -1.83 | -27% | 0.5 | 1.2 | -24% | -29% | -7.01 | 0.01 | -5.42 |
| FDS-3 vs ECRL | -16 | 0.86 | -1.33 | 8% | 0.2 | 15.5 | 29% | 6% | -18.39 | 0.01 | -154.64 |
| FDS-4 vs ECRL | 2 | 0.64 | -2.10 | -16% | 0.9 | 8.3 | 3% | -19% | -21.87 | 0.00 | -168.99 |
| FDS-5 vs ECRL | **-46** | -0.83 | **-3.36** | **-68%** | **2.2** | -3.8 | **-74%** | **-77%** | -12.00 | 0.00 | -103.70 |
| FDP-2 vs ECRL | 22 | 0.42 | -0.96 | -1% | 1.3 | 3.3 | -9% | -21% | -13.76 | -0.03 | -68.33 |
| FDP-3 vs ECRL | -10 | 0.60 | -0.93 | 6% | 1.6 | **20.7** | 22% | 3% | -14.74 | -0.01 | -132.02 |
| FDP-4 vs ECRL | **-39** | 0.42 | -0.57 | 7% | 2.0 | 6.9 | 16% | -10% | -23.47 | 0.00 | -139.55 |
| FDP-5 vs ECRL | -2 | 0.19 | -2.02 | -28% | **3.2** | -1.8 | -24% | **-42%** | -24.34 | -0.01 | -205.50 |
| EIP vs ECRL | **-69** | -0.66 | -1.68 | **-44%** | 0.8 | -16.8 | **-59%** | **-60%** | 5.12 | 0.00 | 53.86 |
| EDC-2 vs ECRL | -13 | -0.71 | -2.47 | **-56%** | 2.2 | -14.0 | **-64%** | **-65%** | -1.73 | 0.00 | -34.66 |
| EDC-3 vs ECRL | **39** | -0.02 | -2.03 | -33% | 1.1 | **-19.5** | -34% | **-36%** | -0.27 | 0.01 | -44.12 |
| EDC-4 vs ECRL | -9 | -0.06 | -2.04 | -33% | **2.6** | -15.3 | **-37%** | **-45%** | -13.12 | 0.04 | -195.26 |
| EDC-5 vs ECRL | **-55** | -0.84 | **-3.22** | **-68%** | **2.9** | -18.5 | **-78%** | **-79%** | 2.02 | 0.02 | -171.90 |
| EDM vs ECRL | **-57** | -0.42 | -2.64 | **-54%** | **2.5** | -9.1 | **-58%** | **-59%** | -0.71 | 0.03 | -112.05 |
| FPL vs ECRL | **-39** | **1.03** | -1.73 | 2% | 0.9 | 0.6 | 13% | -4% | -16.21 | 0.02 | -184.53 |
| APL vs ECRL | **-96** | 0.55 | 0.34 | 29% | 1.2 | -2.0 | 32% | -2% | -26.02 | -0.02 | -101.08 |
| EPB vs ECRL | **-86** | -0.39 | **-2.63** | **-49%** | **3.1** | -4.2 | **-67%** | **-63%** | 27.42 | -0.02 | 183.02 |
| EPL vs ECRL | **-44** | -0.50 | -1.56 | **-39%** | 2.2 | -1.7 | **-51%** | **-58%** | -14.90 | -0.01 | -96.79 |
| ECU vs ECRB | **-39** | -0.17 | -0.04 | -5% | **2.6** | -1.3 | -15% | -19% | -3.32 | -0.01 | -14.10 |
| FDS-2 vs ECRB | 33 | -0.79 | -2.28 | **-49%** | -0.4 | 0.9 | **-54%** | **-46%** | 11.96 | -0.01 | 149.23 |
| FDS-3 vs ECRB | **48** | 0.02 | -1.78 | -25% | -0.8 | 15.1 | -21% | -20% | 0.57 | -0.01 | 0.00 |
| FDS-4 vs ECRB | **66** | -0.21 | **-2.55** | **-41%** | -0.1 | 7.9 | **-37%** | **-39%** | -2.90 | -0.01 | -14.35 |
| FDS-5 vs ECRB | 18 | **-1.67** | **-3.82** | **-77%** | 1.3 | -4.2 | **-84%** | **-83%** | 6.97 | -0.01 | 50.94 |
| FDP-2 vs ECRB | **86** | -0.42 | -1.42 | -31% | 0.3 | 3.0 | **-44%** | **-40%** | 5.20 | -0.04 | 86.31 |
| FDP-3 vs ECRB | **54** | -0.24 | -1.38 | -25% | 0.7 | **20.4** | -25% | -22% | 4.22 | -0.03 | 22.62 |
| FDP-4 vs ECRB | 24 | -0.42 | -1.02 | -25% | 1.0 | 6.5 | -29% | -32% | -4.50 | -0.01 | 15.09 |
| FDP-5 vs ECRB | **62** | -0.65 | **-2.47** | **-49%** | **2.2** | -2.2 | **-54%** | **-56%** | -5.38 | -0.02 | -50.86 |
| EIP vs ECRB | -6 | **-1.50** | -2.13 | **-61%** | -0.1 | -17.1 | **-75%** | **-69%** | 24.08 | -0.01 | 208.51 |
| EDC-2 vs ECRB | **51** | **-1.55** | **-2.92** | **-69%** | 1.2 | -14.3 | **-78%** | **-73%** | 17.23 | -0.01 | 119.99 |
| EDC-3 vs ECRB | **103** | -0.87 | **-2.49** | **-53%** | 0.1 | **-19.9** | **-60%** | **-52%** | 18.69 | -0.01 | 110.53 |
| EDC-4 vs ECRB | **55** | -0.90 | -2.49 | **-53%** | 1.7 | -15.7 | **-62%** | **-59%** | 5.85 | 0.02 | -40.62 |
| EDC-5 vs ECRB | 9 | **-1.69** | **-3.67** | **-78%** | 2.0 | -18.9 | **-86%** | **-84%** | 20.99 | 0.01 | -17.25 |
| EDM vs ECRB | 7 | **-1.26** | **-3.10** | **-67%** | 1.5 | -9.5 | **-74%** | **-69%** | 18.26 | 0.02 | 42.59 |
| FPL vs ECRB | 25 | 0.19 | -2.18 | -29% | -0.1 | 0.3 | -31% | -28% | 2.75 | 0.01 | -29.89 |
| APL vs ECRB | -33 | -0.29 | -0.11 | -9% | 0.2 | -2.4 | -20% | -26% | -7.06 | -0.04 | 53.56 |
| EPB vs ECRB | -23 | **-1.23** | **-3.08** | **-64%** | 2.2 | -4.6 | **-80%** | **-72%** | **46.39** | -0.03 | **337.66** |
| EPL vs ECRB | 19 | **-1.34** | -2.01 | **-57%** | 1.2 | -2.1 | **-70%** | **-69%** | 4.07 | -0.02 | 57.86 |
| FDS-2 vs ECU | **71** | -0.62 | -2.24 | **-46%** | **-3.0** | 2.2 | **-46%** | -34% | 15.28 | 0.00 | 163.33 |
| FDS-3 vs ECU | **86** | 0.19 | -1.75 | -21% | **-3.3** | 16.4 | -7% | -1% | 3.89 | 0.00 | 14.11 |
| FDS-4 vs ECU | **104** | -0.03 | **-2.52** | **-38%** | **-2.6** | 9.2 | -26% | -25% | 0.42 | -0.01 | -0.24 |
| FDS-5 vs ECU | **57** | **-1.50** | **-3.78** | **-76%** | -1.3 | -2.8 | **-81%** | **-79%** | 10.29 | 0.00 | 65.04 |
| FDP-2 vs ECU | **124** | -0.25 | -1.38 | -27% | -2.2 | 4.3 | -35% | -26% | 8.52 | -0.03 | 100.42 |
| FDP-3 vs ECU | **93** | -0.06 | -1.35 | -22% | -1.9 | **21.7** | -12% | -4% | 7.54 | -0.02 | 36.72 |
| FDP-4 vs ECU | **63** | -0.25 | -0.99 | -21% | -1.5 | 7.8 | -17% | -16% | -1.19 | -0.01 | 29.20 |
| FDP-5 vs ECU | **101** | -0.48 | -2.44 | **-47%** | -0.3 | -0.9 | **-46%** | **-46%** | -2.06 | -0.02 | -36.75 |
| EIP vs ECU | 33 | **-1.33** | -2.10 | **-59%** | **-2.7** | -15.8 | **-70%** | **-62%** | 27.40 | -0.01 | 222.61 |
| EDC-2 vs ECU | **90** | **-1.38** | **-2.88** | **-68%** | -1.3 | -13.0 | **-74%** | **-67%** | 20.55 | -0.01 | 134.09 |
| EDC-3 vs ECU | **141** | -0.69 | -2.45 | **-50%** | **-2.4** | -18.6 | **-53%** | **-40%** | 22.01 | 0.00 | 124.63 |
| EDC-4 vs ECU | **94** | -0.72 | -2.45 | **-50%** | -0.9 | -14.4 | **-55%** | **-49%** | 9.16 | 0.03 | -26.52 |
| EDC-5 vs ECU | **48** | **-1.51** | **-3.64** | **-76%** | -0.6 | -17.6 | **-84%** | **-81%** | 24.31 | 0.01 | -3.15 |
| EDM vs ECU | **46** | **-1.08** | **-3.06** | **-66%** | -1.0 | -8.2 | **-70%** | **-62%** | 21.57 | 0.03 | 56.69 |
| FPL vs ECU | **64** | 0.37 | -2.15 | -25% | **-2.6** | 1.6 | -19% | -11% | 6.07 | 0.01 | -15.78 |
| APL vs ECU | 6 | -0.12 | -0.07 | -5% | **-2.3** | -1.1 | -6% | -9% | -3.74 | -0.03 | 67.66 |
| EPB vs ECU | 16 | **-1.06** | **-3.05** | **-62%** | -0.3 | -3.2 | **-76%** | **-65%** | **49.70** | -0.02 | **351.76** |
| EPL vs ECU | **58** | **-1.17** | -1.98 | **-55%** | -1.3 | -0.8 | **-65%** | **-61%** | 7.39 | -0.02 | 71.96 |
| FDS-3 vs FDS-2 | 15 | 0.81 | 0.50 | 48% | -0.3 | 14.3 | **71%** | 49% | -11.38 | 0.00 | -149.22 |
| FDS-4 vs FDS-2 | 33 | 0.59 | -0.27 | 15% | 0.4 | 7.0 | 36% | 13% | -14.86 | -0.01 | -163.57 |
| FDS-5 vs FDS-2 | -14 | -0.88 | -1.54 | **-56%** | 1.7 | -5.0 | **-65%** | **-68%** | -4.99 | 0.00 | -98.29 |
| FDP-2 vs FDS-2 | **53** | 0.37 | 0.86 | 35% | 0.8 | 2.1 | 20% | 11% | -6.75 | -0.03 | -62.91 |
| FDP-3 vs FDS-2 | 21 | 0.55 | 0.90 | 46% | 1.1 | **19.5** | **61%** | 45% | -7.73 | -0.02 | -126.60 |
| FDP-4 vs FDS-2 | -8 | 0.37 | 1.26 | 47% | 1.4 | 5.7 | 53% | 27% | -16.46 | -0.01 | -134.13 |
| FDP-5 vs FDS-2 | 29 | 0.14 | -0.19 | -1% | **2.6** | -3.0 | 0% | -18% | -17.34 | -0.02 | -200.08 |
| EIP vs FDS-2 | **-38** | -0.71 | 0.15 | -23% | 0.3 | -18.0 | **-46%** | **-43%** | 12.13 | -0.01 | 59.28 |
| EDC-2 vs FDS-2 | 19 | -0.76 | -0.64 | **-40%** | 1.7 | -15.2 | **-53%** | **-50%** | 5.28 | -0.01 | -29.24 |
| EDC-3 vs FDS-2 | **70** | -0.08 | -0.21 | -7% | 0.5 | **-20.7** | -13% | -9% | 6.74 | 0.00 | -38.70 |
| EDC-4 vs FDS-2 | 23 | -0.11 | -0.21 | -7% | 2.1 | -16.5 | -17% | -23% | -6.11 | 0.03 | -189.85 |
| EDC-5 vs FDS-2 | -24 | -0.90 | -1.39 | **-56%** | 2.4 | -19.7 | **-71%** | **-71%** | 9.03 | 0.01 | -166.48 |
| EDM vs FDS-2 | -26 | -0.47 | -0.82 | **-36%** | 1.9 | -10.3 | **-45%** | **-43%** | 6.30 | 0.03 | -106.64 |
| FPL vs FDS-2 | -8 | **0.98** | 0.10 | 40% | 0.3 | -0.6 | 49% | 35% | -9.20 | 0.02 | -179.11 |
| APL vs FDS-2 | **-65** | 0.50 | 2.17 | **78%** | 0.7 | -3.3 | **73%** | 38% | -19.01 | -0.03 | -95.67 |
| EPB vs FDS-2 | **-55** | -0.44 | -0.80 | -29% | **2.6** | -5.4 | **-57%** | **-48%** | **34.43** | -0.02 | 188.44 |
| EPL vs FDS-2 | -13 | -0.55 | 0.27 | -16% | 1.6 | -2.9 | **-36%** | **-41%** | -7.89 | -0.02 | -91.37 |
| FDS-4 vs FDS-3 | 18 | -0.22 | -0.77 | -22% | 0.7 | -7.2 | -20% | -24% | -3.47 | -0.01 | -14.35 |
| FDS-5 vs FDS-3 | -29 | **-1.69** | -2.03 | **-70%** | 2.0 | **-19.3** | **-80%** | **-79%** | 6.40 | 0.00 | 50.94 |
| FDP-2 vs FDS-3 | **38** | -0.44 | 0.37 | -8% | 1.1 | -12.1 | -30% | -26% | 4.63 | -0.03 | 86.31 |
| FDP-3 vs FDS-3 | 6 | -0.26 | 0.40 | -1% | 1.4 | 5.3 | -6% | -3% | 3.65 | -0.02 | 22.62 |
| FDP-4 vs FDS-3 | -23 | -0.44 | 0.76 | 0% | 1.8 | -8.6 | -10% | -15% | -5.08 | -0.01 | 15.09 |
| FDP-5 vs FDS-3 | 14 | -0.67 | -0.69 | -33% | **3.0** | -17.3 | **-42%** | **-45%** | -5.95 | -0.02 | -50.86 |
| EIP vs FDS-3 | **-53** | **-1.52** | -0.35 | **-48%** | 0.6 | **-32.2** | **-68%** | **-62%** | 23.51 | -0.01 | 208.50 |
| EDC-2 vs FDS-3 | 4 | **-1.57** | -1.14 | **-59%** | 2.0 | **-29.5** | **-72%** | **-67%** | 16.66 | -0.01 | 119.98 |
| EDC-3 vs FDS-3 | **55** | -0.88 | -0.71 | **-37%** | 0.9 | **-35.0** | **-49%** | **-39%** | 18.12 | 0.00 | 110.52 |
| EDC-4 vs FDS-3 | 8 | -0.92 | -0.71 | **-37%** | **2.5** | **-30.8** | **-51%** | **-49%** | 5.27 | 0.03 | -40.62 |
| EDC-5 vs FDS-3 | -39 | **-1.70** | -1.89 | **-70%** | **2.7** | **-34.0** | **-83%** | **-80%** | 20.41 | 0.01 | -17.26 |
| EDM vs FDS-3 | **-41** | **-1.28** | -1.31 | **-57%** | **2.3** | **-24.6** | **-68%** | **-62%** | 17.68 | 0.03 | 42.59 |
| FPL vs FDS-3 | -23 | 0.17 | -0.40 | -5% | 0.7 | -14.8 | -13% | -10% | 2.18 | 0.01 | -29.89 |
| APL vs FDS-3 | **-80** | -0.31 | 1.67 | 20% | 1.0 | -17.5 | 2% | -8% | -7.63 | -0.03 | 53.55 |
| EPB vs FDS-3 | **-70** | **-1.25** | -1.30 | **-52%** | **3.0** | **-19.7** | **-75%** | **-65%** | **45.81** | -0.02 | **337.66** |
| EPL vs FDS-3 | -28 | **-1.36** | -0.23 | **-43%** | 2.0 | -17.2 | **-63%** | **-61%** | 3.50 | -0.02 | 57.85 |
| FDS-5 vs FDS-4 | **-48** | **-1.47** | -1.26 | **-61%** | 1.3 | -12.0 | **-75%** | **-72%** | 9.87 | 0.00 | 65.29 |
| FDP-2 vs FDS-4 | 20 | -0.22 | 1.14 | 18% | 0.4 | -4.9 | -12% | -2% | 8.10 | -0.03 | 100.66 |
| FDP-3 vs FDS-4 | -12 | -0.03 | 1.17 | 27% | 0.8 | 12.5 | 18% | 28% | 7.12 | -0.01 | 36.97 |
| FDP-4 vs FDS-4 | **-41** | -0.22 | 1.53 | 28% | 1.1 | -1.4 | 12% | 12% | -1.60 | 0.00 | 29.44 |
| FDP-5 vs FDS-4 | -4 | -0.45 | 0.08 | -13% | **2.3** | -10.1 | -27% | -28% | -2.48 | -0.01 | -36.51 |
| EIP vs FDS-4 | **-71** | **-1.30** | 0.42 | -33% | -0.1 | **-25.0** | **-60%** | **-50%** | 26.98 | 0.00 | 222.85 |
| EDC-2 vs FDS-4 | -14 | **-1.35** | -0.37 | **-47%** | 1.3 | **-22.2** | **-65%** | **-56%** | 20.13 | 0.00 | 134.33 |
| EDC-3 vs FDS-4 | **37** | -0.66 | 0.06 | -19% | 0.2 | **-27.8** | **-36%** | -20% | 21.59 | 0.00 | 124.87 |
| EDC-4 vs FDS-4 | -11 | -0.69 | 0.06 | -19% | 1.8 | **-23.6** | **-39%** | -32% | 8.75 | 0.03 | -26.27 |
| EDC-5 vs FDS-4 | **-57** | **-1.48** | -1.12 | **-62%** | 2.0 | **-26.8** | **-79%** | **-74%** | 23.89 | 0.02 | -2.91 |
| EDM vs FDS-4 | **-59** | **-1.05** | -0.54 | **-44%** | 1.6 | -17.4 | **-59%** | **-50%** | 21.16 | 0.03 | 56.93 |
| FPL vs FDS-4 | **-41** | 0.40 | 0.37 | 22% | 0.0 | -7.6 | 9% | 19% | 5.65 | 0.02 | -15.54 |
| APL vs FDS-4 | **-98** | -0.09 | 2.44 | **55%** | 0.3 | -10.3 | 27% | 22% | -4.16 | -0.03 | 67.90 |
| EPB vs FDS-4 | **-88** | **-1.03** | -0.53 | **-39%** | **2.3** | -12.4 | **-68%** | **-54%** | **49.29** | -0.02 | **352.01** |
| EPL vs FDS-4 | **-46** | **-1.14** | 0.54 | -27% | 1.3 | -10.0 | **-53%** | **-48%** | 6.97 | -0.01 | 72.20 |
| FDP-2 vs FDS-5 | **68** | **1.25** | 2.40 | **205%** | -1.0 | 7.1 | **246%** | **246%** | -1.77 | -0.03 | 35.37 |
| FDP-3 vs FDS-5 | 36 | **1.43** | 2.44 | **229%** | -0.6 | **24.5** | **365%** | **352%** | -2.75 | -0.02 | -28.32 |
| FDP-4 vs FDS-5 | 6 | **1.25** | **2.79** | **232%** | -0.3 | 10.7 | **342%** | **295%** | -11.47 | 0.00 | -35.85 |
| FDP-5 vs FDS-5 | **44** | **1.02** | 1.35 | **124%** | 0.9 | 2.0 | **188%** | **154%** | -12.35 | -0.01 | -101.80 |
| EIP vs FDS-5 | -24 | 0.17 | 1.68 | **73%** | -1.4 | -13.0 | **57%** | **77%** | 17.11 | 0.00 | 157.57 |
| EDC-2 vs FDS-5 | 33 | 0.12 | 0.90 | 36% | 0.0 | -10.2 | 37% | **55%** | 10.27 | 0.00 | 69.05 |
| EDC-3 vs FDS-5 | **84** | 0.80 | 1.33 | **109%** | -1.2 | -15.7 | **151%** | **182%** | 11.73 | 0.00 | 59.59 |
| EDC-4 vs FDS-5 | **37** | 0.77 | 1.33 | **108%** | 0.4 | -11.5 | **139%** | **139%** | -1.12 | 0.03 | -91.56 |
| EDC-5 vs FDS-5 | -9 | -0.01 | 0.15 | -1% | 0.7 | -14.7 | -15% | -8% | 14.02 | 0.02 | -68.19 |
| EDM vs FDS-5 | -11 | 0.41 | 0.72 | 44% | 0.2 | -5.3 | **60%** | **78%** | 11.29 | 0.03 | -8.35 |
| FPL vs FDS-5 | 7 | **1.86** | 1.64 | **215%** | -1.4 | 4.4 | **329%** | **319%** | -4.22 | 0.02 | -80.83 |
| APL vs FDS-5 | **-51** | **1.38** | **3.71** | **300%** | -1.0 | 1.7 | **400%** | **329%** | -14.03 | -0.03 | 2.62 |
| EPB vs FDS-5 | **-41** | 0.44 | 0.73 | **59%** | 0.9 | -0.4 | 25% | **63%** | **39.42** | -0.02 | **286.72** |
| EPL vs FDS-5 | 1 | 0.33 | 1.81 | **88%** | -0.1 | 2.1 | **85%** | **83%** | -2.90 | -0.01 | 6.92 |
| FDP-3 vs FDP-2 | -32 | 0.18 | 0.03 | 8% | 0.4 | 17.4 | 34% | 30% | -0.98 | 0.01 | -63.69 |
| FDP-4 vs FDP-2 | **-61** | 0.00 | 0.39 | 9% | 0.7 | 3.6 | 28% | 14% | -9.71 | 0.03 | -71.22 |
| FDP-5 vs FDP-2 | -24 | -0.23 | -1.05 | -26% | 1.9 | -5.1 | -17% | -27% | -10.58 | 0.02 | -137.17 |
| EIP vs FDP-2 | **-91** | **-1.08** | -0.72 | **-43%** | -0.5 | **-20.1** | **-55%** | **-49%** | 18.88 | 0.03 | 122.19 |
| EDC-2 vs FDP-2 | -34 | **-1.13** | -1.50 | **-55%** | 0.9 | -17.3 | **-61%** | **-55%** | 12.03 | 0.03 | 33.67 |
| EDC-3 vs FDP-2 | 17 | -0.45 | -1.07 | -32% | -0.2 | **-22.9** | -27% | -19% | 13.49 | 0.03 | 24.21 |
| EDC-4 vs FDP-2 | -31 | -0.48 | -1.07 | -32% | 1.4 | -18.7 | -31% | -31% | 0.64 | **0.06** | -126.93 |
| EDC-5 vs FDP-2 | **-77** | **-1.26** | -2.25 | **-68%** | 1.6 | **-21.8** | **-76%** | **-74%** | 15.78 | 0.05 | -103.57 |
| EDM vs FDP-2 | **-79** | -0.84 | -1.68 | **-53%** | 1.2 | -12.4 | **-54%** | **-49%** | 13.05 | **0.06** | -43.72 |
| FPL vs FDP-2 | **-61** | 0.61 | -0.76 | 3% | -0.4 | -2.7 | 24% | 21% | -2.45 | 0.05 | -116.20 |
| APL vs FDP-2 | **-118** | 0.13 | 1.31 | 31% | -0.1 | -5.4 | 44% | 24% | -12.26 | 0.00 | -32.76 |
| EPB vs FDP-2 | **-108** | -0.81 | -1.67 | **-48%** | 1.9 | -7.5 | **-64%** | **-53%** | **41.18** | 0.01 | 251.35 |
| EPL vs FDP-2 | **-66** | -0.92 | -0.59 | **-38%** | 0.9 | -5.0 | **-47%** | **-47%** | -1.13 | 0.02 | -28.46 |
| FDP-4 vs FDP-3 | -29 | -0.19 | 0.36 | 1% | 0.3 | -13.8 | -5% | -12% | -8.73 | 0.01 | -7.53 |
| FDP-5 vs FDP-3 | 8 | -0.42 | -1.09 | -32% | 1.5 | **-22.5** | **-38%** | **-44%** | -9.60 | 0.00 | -73.48 |
| EIP vs FDP-3 | **-59** | **-1.27** | -0.75 | **-48%** | -0.8 | **-37.5** | **-66%** | **-61%** | 19.86 | 0.01 | 185.88 |
| EDC-2 vs FDP-3 | -3 | **-1.31** | -1.54 | **-59%** | 0.5 | **-34.7** | **-71%** | **-66%** | 13.01 | 0.02 | 97.37 |
| EDC-3 vs FDP-3 | **49** | -0.63 | -1.11 | **-37%** | -0.6 | **-40.2** | **-46%** | **-38%** | 14.47 | 0.02 | 87.90 |
| EDC-4 vs FDP-3 | 1 | -0.66 | -1.11 | **-37%** | 1.0 | **-36.0** | **-48%** | **-47%** | 1.62 | 0.05 | -63.24 |
| EDC-5 vs FDP-3 | **-45** | **-1.45** | -2.29 | **-70%** | 1.3 | **-39.2** | **-82%** | **-80%** | 16.76 | 0.03 | -39.88 |
| EDM vs FDP-3 | **-47** | -1.02 | -1.71 | **-56%** | 0.8 | **-29.8** | **-66%** | **-61%** | 14.03 | 0.05 | 19.97 |
| FPL vs FDP-3 | -29 | 0.43 | -0.80 | -4% | -0.8 | **-20.1** | -8% | -7% | -1.47 | 0.04 | -52.51 |
| APL vs FDP-3 | **-86** | -0.05 | 1.27 | 22% | -0.5 | **-22.8** | 8% | -5% | -11.28 | -0.01 | 30.94 |
| EPB vs FDP-3 | **-76** | **-0.99** | -1.70 | **-52%** | 1.5 | **-24.9** | **-73%** | **-64%** | **42.16** | 0.00 | **315.04** |
| EPL vs FDP-3 | -34 | **-1.10** | -0.63 | **-43%** | 0.5 | **-22.4** | **-60%** | **-60%** | -0.15 | 0.00 | 35.23 |
| FDP-5 vs FDP-4 | **38** | -0.23 | -1.45 | -32% | 1.2 | -8.7 | **-35%** | **-36%** | -0.87 | -0.01 | -65.95 |
| EIP vs FDP-4 | -30 | **-1.08** | -1.11 | **-48%** | -1.1 | **-23.7** | **-65%** | **-55%** | 28.59 | 0.00 | 193.41 |
| EDC-2 vs FDP-4 | 27 | **-1.13** | -1.90 | **-59%** | 0.2 | **-20.9** | **-69%** | **-61%** | 21.74 | 0.00 | 104.89 |
| EDC-3 vs FDP-4 | **78** | -0.44 | -1.46 | **-37%** | -0.9 | **-26.4** | **-43%** | -29% | 23.20 | 0.01 | 95.43 |
| EDC-4 vs FDP-4 | 31 | -0.47 | -1.47 | **-37%** | 0.7 | **-22.2** | **-46%** | **-39%** | 10.35 | 0.04 | -55.71 |
| EDC-5 vs FDP-4 | -16 | **-1.26** | -2.65 | **-70%** | 0.9 | **-25.4** | **-81%** | **-77%** | 25.49 | 0.02 | -32.35 |
| EDM vs FDP-4 | -18 | -0.83 | -2.07 | **-57%** | 0.5 | -16.0 | **-64%** | **-55%** | 22.76 | 0.04 | 27.50 |
| FPL vs FDP-4 | 1 | 0.62 | -1.16 | -5% | -1.1 | -6.3 | -3% | 6% | 7.26 | 0.02 | -44.98 |
| APL vs FDP-4 | **-57** | 0.13 | 0.91 | 21% | -0.8 | -9.0 | 13% | 9% | -2.55 | -0.02 | 38.46 |
| EPB vs FDP-4 | **-47** | -0.81 | -2.06 | **-52%** | 1.2 | -11.1 | **-72%** | **-59%** | **50.89** | -0.02 | **322.57** |
| EPL vs FDP-4 | -5 | -0.92 | -0.99 | **-43%** | 0.2 | -8.6 | **-58%** | **-54%** | 8.58 | -0.01 | 42.76 |
| EIP vs FDP-5 | **-68** | -0.85 | 0.34 | -23% | **-2.3** | -15.0 | **-46%** | -30% | 29.46 | 0.01 | **259.36** |
| EDC-2 vs FDP-5 | -11 | -0.90 | -0.45 | **-39%** | -1.0 | -12.2 | **-53%** | **-39%** | 22.61 | 0.01 | 170.84 |
| EDC-3 vs FDP-5 | **41** | -0.21 | -0.02 | -7% | -2.1 | -17.7 | -13% | 11% | 24.07 | 0.02 | 161.38 |
| EDC-4 vs FDP-5 | -7 | -0.25 | -0.02 | -7% | -0.5 | -13.5 | -17% | -6% | 11.22 | 0.04 | 10.24 |
| EDC-5 vs FDP-5 | **-53** | **-1.03** | -1.20 | **-56%** | -0.3 | -16.7 | **-71%** | **-64%** | 26.37 | 0.03 | 33.60 |
| EDM vs FDP-5 | **-55** | -0.60 | -0.63 | **-36%** | -0.7 | -7.3 | **-45%** | -30% | 23.63 | 0.04 | 93.45 |
| FPL vs FDP-5 | **-37** | 0.84 | 0.29 | 40% | **-2.3** | 2.4 | 49% | **65%** | 8.13 | 0.03 | 20.97 |
| APL vs FDP-5 | **-94** | 0.36 | 2.36 | **79%** | -2.0 | -0.2 | **74%** | **69%** | -1.68 | -0.02 | 104.41 |
| EPB vs FDP-5 | **-84** | -0.58 | -0.61 | -29% | 0.0 | -2.4 | **-57%** | **-36%** | **51.76** | -0.01 | **388.52** |
| EPL vs FDP-5 | **-43** | -0.69 | 0.46 | -16% | -1.0 | 0.1 | **-36%** | -28% | 9.45 | 0.00 | 108.71 |
| EDC-2 vs EIP | **57** | -0.05 | -0.79 | -21% | 1.4 | 2.8 | -13% | -13% | -6.85 | 0.00 | -88.52 |
| EDC-3 vs EIP | **108** | 0.64 | -0.36 | 21% | 0.2 | -2.8 | **60%** | **59%** | -5.39 | 0.01 | -97.98 |
| EDC-4 vs EIP | **61** | 0.60 | -0.36 | 21% | 1.8 | 1.4 | 53% | 35% | -18.24 | 0.03 | **-249.13** |
| EDC-5 vs EIP | 14 | -0.18 | -1.54 | **-43%** | 2.1 | -1.7 | **-46%** | **-48%** | -3.10 | 0.02 | -225.76 |
| EDM vs EIP | 13 | 0.25 | -0.96 | -17% | 1.6 | 7.7 | 2% | 0% | -5.83 | 0.03 | -165.92 |
| FPL vs EIP | 31 | **1.69** | -0.05 | **82%** | 0.1 | 17.4 | **173%** | **136%** | -21.33 | 0.02 | -238.39 |
| APL vs EIP | -27 | **1.21** | 2.02 | **132%** | 0.4 | 14.7 | **219%** | **142%** | **-31.14** | -0.03 | -154.95 |
| EPB vs EIP | -17 | 0.27 | -0.95 | -8% | **2.3** | 12.6 | -21% | -8% | 22.30 | -0.02 | 129.16 |
| EPL vs EIP | 25 | 0.16 | 0.12 | 9% | 1.4 | 15.0 | 18% | 3% | -20.01 | -0.01 | -150.65 |
| EDC-3 vs EDC-2 | **51** | 0.68 | 0.43 | **53%** | -1.1 | -5.5 | **84%** | **83%** | 1.46 | 0.00 | -9.46 |
| EDC-4 vs EDC-2 | 4 | 0.65 | 0.43 | 53% | 0.5 | -1.3 | **75%** | **55%** | -11.39 | 0.03 | -160.61 |
| EDC-5 vs EDC-2 | **-42** | -0.14 | -0.75 | -27% | 0.7 | -4.5 | **-38%** | **-41%** | 3.75 | 0.02 | -137.24 |
| EDM vs EDC-2 | **-44** | 0.29 | -0.18 | 6% | 0.3 | 4.9 | 17% | 15% | 1.02 | 0.03 | -77.40 |
| FPL vs EDC-2 | -26 | **1.74** | 0.74 | **131%** | -1.3 | 14.6 | **214%** | **171%** | -14.48 | 0.02 | -149.87 |
| APL vs EDC-2 | **-84** | **1.26** | **2.81** | **194%** | -1.0 | 11.9 | **266%** | **178%** | -24.29 | -0.03 | -66.43 |
| EPB vs EDC-2 | **-74** | 0.32 | -0.16 | 17% | 1.0 | 9.8 | -9% | 6% | 29.15 | -0.02 | 217.67 |
| EPL vs EDC-2 | -32 | 0.21 | 0.91 | 39% | 0.0 | 12.3 | 35% | 18% | -13.16 | -0.01 | -62.13 |
| EDC-4 vs EDC-3 | **-48** | -0.03 | 0.00 | 0% | 1.6 | 4.2 | -5% | -15% | -12.85 | 0.03 | -151.15 |
| EDC-5 vs EDC-3 | **-94** | -0.82 | -1.18 | **-53%** | 1.8 | 1.0 | **-66%** | **-67%** | 2.29 | 0.01 | -127.78 |
| EDM vs EDC-3 | **-96** | -0.39 | -0.61 | -31% | 1.4 | 10.4 | **-36%** | **-37%** | -0.44 | 0.03 | -67.94 |
| FPL vs EDC-3 | **-78** | **1.06** | 0.31 | 51% | -0.2 | **20.2** | **71%** | 49% | -15.94 | 0.02 | -140.41 |
| APL vs EDC-3 | **-135** | 0.58 | 2.38 | **92%** | 0.1 | 17.5 | **99%** | **52%** | -25.75 | -0.03 | -56.97 |
| EPB vs EDC-3 | **-125** | -0.37 | -0.59 | -24% | 2.1 | 15.3 | **-50%** | **-42%** | 27.69 | -0.02 | 227.14 |
| EPL vs EDC-3 | **-83** | -0.47 | 0.48 | -10% | 1.1 | 17.8 | -27% | **-35%** | -14.62 | -0.02 | -52.67 |
| EDC-5 vs EDC-4 | **-46** | -0.79 | -1.18 | **-53%** | 0.3 | -3.2 | **-65%** | **-62%** | 15.14 | -0.02 | 23.37 |
| EDM vs EDC-4 | **-48** | -0.36 | -0.61 | -31% | -0.2 | 6.2 | -33% | -26% | 12.41 | 0.00 | 83.21 |
| FPL vs EDC-4 | -30 | **1.09** | 0.31 | 51% | -1.8 | 16.0 | **79%** | **75%** | -3.09 | -0.01 | 10.73 |
| APL vs EDC-4 | **-88** | 0.61 | 2.38 | **92%** | -1.5 | 13.3 | **109%** | **80%** | -12.90 | **-0.06** | 94.18 |
| EPB vs EDC-4 | **-78** | -0.33 | -0.59 | -24% | 0.5 | 11.1 | **-48%** | -32% | **40.54** | -0.05 | **378.28** |
| EPL vs EDC-4 | -36 | -0.44 | 0.48 | -10% | -0.5 | 13.6 | -23% | -24% | -1.77 | -0.04 | 98.48 |
| EDM vs EDC-5 | -2 | 0.43 | 0.57 | 46% | -0.4 | 9.4 | **89%** | **94%** | -2.73 | 0.01 | 59.84 |
| FPL vs EDC-5 | 16 | **1.88** | 1.49 | **219%** | -2.0 | 19.1 | **407%** | **357%** | -18.23 | 0.00 | -12.63 |
| APL vs EDC-5 | **-41** | **1.40** | **3.56** | **305%** | -1.7 | 16.5 | **492%** | **368%** | -28.04 | -0.04 | 70.81 |
| EPB vs EDC-5 | -31 | 0.45 | 0.59 | **61%** | 0.3 | 14.3 | 47% | **78%** | 25.40 | -0.04 | **354.92** |
| EPL vs EDC-5 | 11 | 0.35 | 1.66 | **91%** | -0.7 | 16.8 | **118%** | **99%** | -16.92 | -0.03 | 75.11 |
| FPL vs EDM | 18 | **1.45** | 0.92 | **119%** | -1.6 | 9.7 | **168%** | **136%** | -15.50 | -0.01 | -72.47 |
| APL vs EDM | **-39** | 0.97 | **2.99** | **178%** | -1.3 | 7.1 | **213%** | **142%** | -25.31 | **-0.06** | 10.97 |
| EPB vs EDM | -29 | 0.03 | 0.01 | 11% | 0.7 | 4.9 | -22% | -8% | 28.13 | -0.05 | **295.07** |
| EPL vs EDM | 13 | -0.08 | 1.08 | 31% | -0.3 | 7.4 | 16% | 3% | -14.18 | -0.04 | 15.27 |
| APL vs FPL | **-58** | -0.48 | 2.07 | 27% | 0.3 | -2.7 | 17% | 2% | -9.81 | -0.05 | 83.44 |
| EPB vs FPL | **-48** | **-1.42** | -0.90 | **-49%** | **2.3** | -4.8 | **-71%** | **-61%** | **43.63** | -0.04 | **367.55** |
| EPL vs FPL | -6 | **-1.53** | 0.17 | **-40%** | 1.3 | -2.4 | **-57%** | **-56%** | 1.32 | -0.03 | 87.74 |
| EPB vs APL | 10 | -0.94 | **-2.97** | **-60%** | 2.0 | -2.1 | **-75%** | **-62%** | **53.44** | 0.01 | **284.10** |
| EPL vs APL | **52** | **-1.05** | -1.90 | **-53%** | 1.0 | 0.3 | **-63%** | **-57%** | 11.13 | 0.01 | 4.30 |
| EPL vs EPB | **42** | -0.11 | 1.07 | 19% | -1.0 | 2.5 | 48% | 12% | **-42.31** | 0.01 | **-279.81** |
| DTT vs SSP |  |  |  |  | **-4.6** | -2.1 |  |  |  |  |  |
| DTT vs IS |  |  |  |  | -1.9 | 5.0 |  |  |  |  |  |
| DTT vs SSC |  |  |  |  | -1.9 | 0.1 |  |  |  |  |  |
| DTT vs LHBT |  |  |  |  | -0.7 | -14.5 |  |  |  |  |  |
| DTT vs DBT |  |  |  |  | 1.4 | -11.6 |  |  |  |  |  |
| DTT vs BrR |  |  |  |  | 1.8 | -10.2 |  |  |  |  |  |
| FCR vs DTT |  |  |  |  | -2.0 | 17.1 |  |  |  |  |  |
| PL vs DTT |  |  |  |  | -1.4 | 17.6 |  |  |  |  |  |
| FCU vs DTT |  |  |  |  | -1.9 | 16.6 |  |  |  |  |  |
| ECRL vs DTT |  |  |  |  | -1.7 | **19.2** |  |  |  |  |  |
| ECRB vs DTT |  |  |  |  | -0.7 | **19.5** |  |  |  |  |  |
| ECU vs DTT |  |  |  |  | 1.8 | 18.2 |  |  |  |  |  |
| FDS-2 vs DTT |  |  |  |  | -1.1 | **20.4** |  |  |  |  |  |
| FDS-3 vs DTT |  |  |  |  | -1.5 | **34.6** |  |  |  |  |  |
| FDS-4 vs DTT |  |  |  |  | -0.8 | **27.4** |  |  |  |  |  |
| FDS-5 vs DTT |  |  |  |  | 0.6 | 15.4 |  |  |  |  |  |
| FDP-2 vs DTT |  |  |  |  | -0.4 | **22.5** |  |  |  |  |  |
| FDP-3 vs DTT |  |  |  |  | 0.0 | **39.9** |  |  |  |  |  |
| FDP-4 vs DTT |  |  |  |  | 0.3 | **26.1** |  |  |  |  |  |
| FDP-5 vs DTT |  |  |  |  | 1.5 | 17.4 |  |  |  |  |  |
| EIP vs DTT |  |  |  |  | -0.9 | 2.4 |  |  |  |  |  |
| EDC-2 vs DTT |  |  |  |  | 0.5 | 5.2 |  |  |  |  |  |
| EDC-3 vs DTT |  |  |  |  | -0.6 | -0.4 |  |  |  |  |  |
| EDC-4 vs DTT |  |  |  |  | 1.0 | 3.8 |  |  |  |  |  |
| EDC-5 vs DTT |  |  |  |  | 1.2 | 0.7 |  |  |  |  |  |
| EDM vs DTT |  |  |  |  | 0.8 | 10.1 |  |  |  |  |  |
| FPL vs DTT |  |  |  |  | -0.8 | **19.8** |  |  |  |  |  |
| APL vs DTT |  |  |  |  | -0.5 | 17.1 |  |  |  |  |  |
| EPB vs DTT |  |  |  |  | 1.5 | 15.0 |  |  |  |  |  |
| EPL vs DTT |  |  |  |  | 0.5 | 17.4 |  |  |  |  |  |

Appendix References

1. Ashton DM, Blaker CL, Hartnell N, et al. Challenging the Perceptions of Human Tendon Allografts: Influence of Donor Age, Sex, Height, and Tendon on Biomechanical Properties. *Am J Sports Med.* 2023;51(3):768-778.

2. Ashton DM, Blaker CL, Hartnell N, et al. The Biomechanical, Biochemical, and Morphological Properties of 19 Human Cadaveric Lower Limb Tendons and Ligaments: An Open-Access Data Set. *Am J Sports Med.* 2024;52(9):2391-2401.

3. Bentley JP, Hanson AN. The hydroxyproline of elastin. *Biochim Biophys Acta.* 1969;175(2):339-344.

4. [Dataset] Blaker CL, Ashton DM, Hefferan S, Little CB, Clarke EC. Dataset 2 for: "The biomechanical, biochemical and morphological properties of 31 human cadaveric upper limb tendons: an open access dataset part II". 2025. doi:10.7910/DVN/LXN3EH.

5. Dimitriou I, Katsourakis A, Natsis K, Kostretzis L, Noussios G. Palmaris longus muscle’s prevalence in different nations and interesting anatomical variations: review of the literature. *J Clin Med Res.* 2015;7(11):825.

6. Keener JD, Chafik D, Kim HM, Galatz LM, Yamaguchi K. Insertional anatomy of the triceps brachii tendon. *J Shoulder Elbow Surg.* 2010;19(3):399-405.

7. Kharaz YA, Canty-Laird EG, Tew SR, Comerford EJ. Variations in internal structure, composition and protein distribution between intra- and extra-articular knee ligaments and tendons. *J Anat.* 2018;232(6):943-955.

8. Neuman RE, Logan MA. The determination of hydroxyproline. *The Journal of biological chemistry.* 1950;184(1):299-306.

9. Townley WA, Swan MC, Dunn RL. Congenital absence of flexor digitorum superficialis: implications for assessment of little finger lacerations. *J Hand Surg Eur Vol.* 2010;35(5):417-418.

10. Zilber S, Oberlin C. Anatomical variations of the extensor tendons to the fingers over the dorsum of the hand: a study of 50 hands and a review of the literature. *Plast Reconstr Surg.* 2004;113(1):214-221.
